# Effectiveness of Percutaneous Needle Electrolysis (PNE) and Intramuscular Electrical Stimulation (IMES) in the Management of Myofascial Pain Syndrome and Tendinopathies: A Systematic Review

**DOI:** 10.3390/jcm15072572

**Published:** 2026-03-27

**Authors:** Robert Trybulski, Gracjan Olaniszyn, Małgorzata Smoter, Olha Bas, Oksana Tyravska, Michał Kuszewski, Katarzyna Walicka-Cupryś

**Affiliations:** 1 Department of Medical Sciences, Faculty of Medical Sciences, Upper Silesian Academy of Wojciech Korfanty in Katowice, 40-659 Katowice, Poland; 2 Provita Medical Centre, 44-240 Żory, Poland; 3 Olaniszyn Physiotherapy Centre, 47-400 Racibórz, Poland; 4 Department of Basic Physiotherapy, Gdansk University of Physical Education and Sport, 80-336 Gdansk, Poland; 5 Department of Therapy and Rehabilitation, Faculty of Therapy and Rehabilitation, Ivan Boberskyi Lviv State University of Physical Culture, 79000 Lviv, Ukraine; 6 Institute of Physioterapy and Health Sciences, Academy of Physical Education, 40-065 Katowice, Poland; 7 Faculty of Health Sciences and Psychology, Institute of Physiotherapy, University of Rzeszów, ul. Al. T. Rejtana 19 C, 35-959 Rzeszów, Poland

**Keywords:** percutaneous needle electrolysis, intramuscular electrical stimulation, myofascial pain syndrome, tendinopathies

## Abstract

**Objectives**: Myofascial pain syndrome (MPS) is a common musculoskeletal condition, and while percutaneous needle electrolysis (PNE) and intramuscular electrical stimulation (IMES) are emerging therapies for myofascial pain syndrome and tendinopathies, their effects remain unclear. This systematic review aimed to characterize the methodological features and synthesize the evidence on the clinical improvement and adverse events rates of PNE and IMES in treating MPS and tendinopathies. Data Sources: PubMed, Scopus, Web of Science, ClinicalTrials.gov, the World Health Organization International Clinical Trials Registry Platform, Google Scholar, and reference lists. Searches were carried out on 10 July 2025 and repeated on 16 March 2026, just before final analysis. New results found during final searches were screened for inclusion to ensure currency of the review. **Methods**: We selected studies based on the PICOS framework and predefined selection criteria: Population: adults with MPS or active myofascial trigger points (TrPs), or tendinopathies; Intervention: PNE or IMES; Comparator: sham procedures, other interventions, or no intervention; Outcomes: pain intensity (e.g., Visual Analogue Scale or Numeric Pain Rating Scale), pressure pain threshold (PPT), and functional measures; and Study Design: experimental studies. Studies focused exclusively on post-surgical or neuropathic pain, studies without a relevant comparator, and studies not reporting clinically meaningful outcomes were excluded. We assessed the risk of bias of included studies and performed a narrative synthesis. **Results**: From 737 identified records, 30 studies met the selection criteria. PNE was generally effective in reducing pain and improving function in tendinopathies and MPS, although results varied across outcomes and follow-ups. IMES showed moderate evidence for reducing pain and enhancing function, particularly cervical range of motion and PPT. However, both interventions had inconsistent clinical improvement and adverse events rates on disability indices and quality of life. Most studies had a high risk of bias due to challenges in blinding. Reported adverse events were minor and self-limiting, indicating that both therapies are generally safe when performed by trained clinicians. **Conclusions**: PNE and IMES may improve pain and some functional outcomes in MPS and tendinopathies; however, these findings should be interpreted cautiously because most included studies had a high risk of bias.

## 1. Introduction

Myofascial pain syndrome (MPS) is one of the most prevalent musculoskeletal disorders, characterized by the presence of painful myofascial trigger points (TrPs) within taut bands of skeletal muscle fibers. These trigger points can generate both localized and referred pain, limited range of motion, muscle stiffness, and impaired motor function [1]. The etiology of MPS is multifactorial, involving biomechanical, neurological, psychosocial, and ischemic factors [2]. Due to its often-chronic nature and diagnostic challenges, MPS poses a significant therapeutic and economic burden.

It is estimated that MPS affects between 30% and 85% of patients seeking care for musculoskeletal pain, with chronic forms substantially impacting quality of life, work ability, and healthcare utilization [3,4]. Epidemiological analyses indicate that the indirect costs associated with sick leave, reduced productivity, and prolonged pharmacological or physiotherapy treatment are substantial. In the United States alone, the annual costs of managing musculoskeletal pain syndromes, including MPS, exceed $100 billion, with a large proportion attributed to indirect expenses [5].

While traditional approaches, including pharmacotherapy, manual therapy, and invasive procedures, remain widely used, they often yield only partial or temporary relief and carry the risk of adverse effects. Emerging evidence supports the effectiveness of multimodal interventions, especially those integrating dry needling, trigger point therapy [6], exercise therapy [7], transcutaneous electrical nerve stimulation (TENS) [8], and acupuncture [9]. Among these, minimally invasive electrotherapeutic techniques such as percutaneous needle electrolysis (PNE) [10] and intramuscular electrical stimulation (IMES) [11] have gained attention. Therefore, the development of effective, safe, and cost-efficient treatment strategies—such as PNE and IMES—has important clinical and societal implications [12].

PNE involves the application of galvanic current via acupuncture needles into soft tissues [13], while intramuscular electrical stimulation (IMES) is a needling-based electrotherapeutic technique in which a low-frequency electrical current is delivered through inserted needles directly into muscle tissue, typically targeting myofascial trigger points (TrPs) or related neuromuscular structures [14]. Unlike transcutaneous electrical nerve stimulation (TENS), which delivers current through surface electrodes placed on the skin to stimulate peripheral nerves non-invasively, IMES applies the current intramuscularly and therefore targets deeper tissues more focally [15]. IMES should also be distinguished from electroacupuncture, in which electrical stimulation is applied through acupuncture needles inserted at specific acupuncture points; although the procedures may appear technically similar, the main distinction lies in the therapeutic rationale and anatomical target, with electroacupuncture being based primarily on acupoint stimulation, whereas IMES is directed toward muscle tissue, TrPs, or motor points to modulate neuromuscular dysfunction [16]. Thus, IMES refers specifically to electrical stimulation delivered through needles inserted into muscle tissue/trigger points, rather than transcutaneous stimulation (TENS) or electroacupuncture applied according to traditional acupoint selection [14]. These interventions are thought to influence peripheral and central pain pathways and are grounded in mechanisms described by the Gate Control Theory of Pain [17]. Their minimally invasive nature makes them attractive alternatives to more aggressive or systemic interventions [18]. Although some studies report promising results for PNE [10] and IMES [19] in managing MPS and related conditions, the clinical evidence remains inconsistent due to wide variations in treatment protocols, such as frequency, duration, current intensity, and anatomical targets.

A systematic review published in 2020 [20] dedicated to assessing the effects of ultrasound-guided PNE for musculoskeletal pain verified that, based on moderate-quality evidence, PNE significantly reduced pain intensity and pain-related disability across short-, mid-, and long-term follow-ups, although heterogeneity among studies slightly downgraded the overall evidence strength. In 2022, a review [18] dedicated to evaluating the clinical use of PNE in musculoskeletal conditions also verified that, despite moderate short- and mid-term effects on pain relief, the overall evidence remains limited due to methodological weaknesses, heterogeneous treatment protocols, and a lack of consistent findings regarding functional or structural improvements. Considering IMES, a review [11] revealed that, based on six randomized controlled trials involving 158 patients with myofascial pain syndrome, IMES was generally more effective than sham-IMES, dry needling, or exercise therapy in improving pain, pressure pain threshold, range of motion, and reducing analgesic use, though the evidence remains preliminary and limited by small sample sizes.

Although a growing body of research supports the use of PNE and, to a lesser extent, IMES, recent evidence indicates that PNE may reduce pain and disability in tendinopathies, while tendinopathy-specific evidence for IMES remains limited and inconsistent [21,22,23]. Randomized trials in Achilles and supraspinatus tendinopathies have reported favorable outcomes for PNE, particularly when combined with eccentric exercise or multimodal rehabilitation [24,25]. In contrast, intramuscular stimulation did not provide additional benefit over exercise-based rehabilitation in chronic midportion Achilles tendinopathy, underscoring the more limited and uncertain evidence base for IMES in tendinopathy [26]. As a result, clinicians still lack an integrated, evidence-based synthesis comparing the effectiveness, safety, and clinical indications of PNE and IMES across both MPS and tendinopathies.

This systematic review addressed the following questions: (1) in adults with myofascial pain syndrome, what are the clinical improvement and adverse events rates of PNE versus comparators and IMES versus comparators; and (2) in adults with tendinopathy, what are the clinical improvement and adverse events rates of PNE versus comparators and IMES versus comparators? Based on such questions we aimed to evaluate the effects of percutaneous needle electrolysis (PNE) and intramuscular electrical stimulation (IMES) in adults with myofascial pain syndrome or tendinopathies. Randomized and quasi-randomized clinical trials comparing these interventions with sham procedures, placebo, dry needling, conventional physical therapy, or other standard care were included. The primary outcomes were pain intensity, functional improvement, and neuromuscular performance; secondary outcomes included pressure pain threshold, patient-reported outcomes, safety, and adverse events. Results were reported separately for each intervention-condition pairing.

## 2. Materials and Methods

This systematic review was conducted in accordance with the PRISMA 2020 Statement (Preferred Reporting Items for Systematic Reviews and Meta-Analyses) [27]. The protocol was registered in the International Platform of Registered Systematic Review and Meta-Analysis Protocols (Date of registration: 11 July 2025; INPLASY202570048). No modifications were made regarding the published protocol.

### 2.1. Eligibility Criteria

The review question was structured using the PICOS framework. Eligibility criteria were prespecified in terms of participants, interventions, comparators, outcomes, study design, and report characteristics.

Population: Eligible studies included adults and adolescents (≥16 years) with myofascial pain syndrome (MPS), localized muscle pain with active or latent myofascial trigger points (TrPs), or tendinopathies. Only studies in which one of these conditions was the primary diagnosis under investigation were included. Studies focused exclusively on post-surgical pain, neuropathic pain, or other clinical conditions were excluded unless MPS or tendinopathy was clearly identified as the main condition of interest. This criterion was established to ensure consistency with the review objective and to maintain focus on the specific clinical entities in which PNE and IMES are most relevant.

Intervention: Eligible interventions included percutaneous needle electrolysis (PNE) or intramuscular electrical stimulation (IMES), applied either alone or in combination with complementary interventions such as therapeutic exercise or manual therapy. PNE was considered eligible when applied to the symptomatic musculoskeletal tissue relevant to the target condition, including myofascial trigger points or related muscle tissue in myofascial pain syndrome, and the tendon or peritendinous region in tendinopathies. IMES was considered eligible when electrical stimulation was delivered through inserted needles to muscle tissue, trigger points, or related neuromuscular targets. When PNE or IMES were delivered as part of a combined intervention, studies were considered eligible only if the additional complementary treatment was applied under similar conditions in both comparison arms (e.g., the same therapeutic exercise program in both groups), thereby allowing the specific contribution of PNE or IMES to be meaningfully evaluated. These interventions represent the focus of this review, aiming to consolidate evidence on their direct effects. Only PNE and IMES were eligible as primary interventions. Studies labeled by the original authors as percutaneous electrical nerve stimulation PENS were included only when the technique involved needle-based delivery of electrical current to muscular or myofascial trigger-point targets and was therefore classified in this review as an IMES-type intervention. Standalone percutaneous neuromodulation/manipulation techniques not meeting these criteria were excluded. This criterion was adopted to ensure conceptual relevance to the neuromuscular effects proposed for PNE and IMES, while distinguishing these approaches from broader nerve-targeted stimulation techniques.

Comparators: Comparator interventions included a range of control or alternative treatment approaches: sham procedures, placebo, dry needling, conventional physical therapy, pharmacological treatments, or no intervention/standard care approaches. Studies were excluded if they did not include a comparator arm (either passive or active), or if the comparator intervention was not relevant to the clinical management of MPS or tendinopathies.

Outcomes: Studies were included if they reported on any of the specified primary or secondary clinical outcomes. Primary outcomes of interest were pain intensity (e.g., measured by Visual Analogue Scale (VAS) or Numeric Pain Rating Scale (NPRS)), functional improvement (e.g., assessed by Range of Motion (ROM) or disability indices), and neuromuscular performance. These primary outcomes were selected as they directly reflect the patient’s experience, ability to perform daily activities, and objective physiological changes, which are central to assessing the effectiveness of the interventions. Secondary outcomes included safety profiles, the incidence and nature of adverse effects, changes in pressure pain threshold (PPT), and other patient-reported outcomes; these provide a holistic view of the interventions’ impact, including potential risks and patient perspectives. Studies that did not include these clinical endpoints, or those relying solely on imaging or biomarker data without corresponding clinical measures, were explicitly excluded to ensure the review focuses on clinically meaningful results and direct patient benefit.

Study Design: Considered study designs included randomized experimental and controlled studies (e.g., randomized controlled trials [RCTs]) and non-randomized experimental and controlled studies (e.g., quasi-randomized clinical trials, controlled clinical trials [CCTs]). All included studies were required to feature a comparator arm to allow for the assessment of intervention effectiveness and to minimize bias. Reviews, case reports, and studies for which the full text was unavailable were excluded due to their inherent limitations in providing primary, unbiased data suitable for systematic synthesis.

### 2.2. Information Sources

The search strategy was systematically conducted across multiple electronic databases and registers to ensure comprehensive identification of relevant studies. Bibliographic databases searched included PubMed, Scopus, and Web of Science. Searches were carried out on 10 July 2025 and repeated on 16 March 2026, just before final analysis. New results found during final searches were screened for inclusion to ensure currency of the review The dates of coverage for these databases extended from their inception up to the last search date.

In addition to bibliographic databases, clinical trial registers were consulted to identify ongoing or unpublished studies and to minimize publication bias. These included ClinicalTrials.gov and the World Health Organization International Clinical Trials Registry Platform (WHO ICTRP). These registers were also searched up to 10 June 2025, with no date restrictions applied within their respective coverages.

Complementary search methods included browsing Google Scholar to identify additional relevant literature and gray literature. This supplementary search was also completed on 10 June 2025. In addition to database and Google Scholar searches, a targeted Google search was performed to identify grey literature and scholarly articles not indexed in bibliographic databases. Simplified search strings derived from the main database strategy were used, and the first 100 results for each search string were screened. Furthermore, manual reference checking was performed by examining the reference lists of all included studies and relevant systematic reviews identified during the database searches. This process aimed to identify any potentially eligible studies not captured by the electronic searches. The manual reference checking was concluded on 10 June 2025. No direct contact with organizations, manufacturers, or individual authors was conducted to identify studies for this review.

### 2.3. Search Strategy

The search strategy was developed using a combination of Medical Subject Headings (MeSH) terms and keywords related to the population, intervention, and condition of interest, adapting the strategy for each specific database’s syntax and search functionalities. Searches were carried out on 10 July 2025 and repeated on 16 March 2026, just before final analysis. New results found during final searches were screened for inclusion to ensure currency of the review. Table 1 shows the search strategy used for PubMed, Scopus, and Web of Science Core Collection.

No filters were applied regarding publication date or language in the electronic searches. This approach aims to minimize publication bias and ensure a comprehensive retrieval of all potentially relevant evidence. No specific search filters designed to retrieve particular record types were adopted from published approaches. Additionally, no natural language processing or text frequency analysis tools were used for keyword refinement, nor were any tools utilized for automatic translation of search strings or for search strategy validation or peer review via checklists such as PRESS.

### 2.4. Selection Process

The study selection process was conducted systematically and independently by two authors at each stage. Titles and abstracts of all records retrieved from the searches were initially screened against the predefined eligibility criteria. Subsequently, the full texts of potentially relevant articles were retrieved and independently assessed for eligibility by the same two authors. Any disagreements that arose during either the title/abstract screening or full-text review stages were resolved through discussion between the two authors; in cases where consensus could not be reached, a third author was consulted to arbitrate and make the final decision. Duplicates across all databases were identified and removed using EndNote (version 20.5) prior to the initiation of the screening process. This review did not utilize any automation tools (e.g., machine learning classifiers) for study selection, nor did it involve crowdsourcing or rely on previously screened datasets. All screening decisions were made manually by the independent authors. No abstracts or articles required translation into another language solely to determine their eligibility; all pertinent information was available in English for this purpose.

### 2.5. Data Collection

Data extraction from the included studies was performed independently by two authors using a standardized Excel form. This form was designed to systematically capture key information, including study characteristics (e.g., design, sample size), detailed population demographics and clinical details (e.g., diagnosis, age range, activity level), intervention and comparator protocols (e.g., specific parameters, duration, frequency), outcome measures and their respective time points, follow-up duration, and markers relevant for methodological quality assessment. Any discrepancies identified during data extraction were resolved through discussion between the two authors. In instances of missing or unclear data necessary for the review, authors of the respective studies were contacted via email to obtain clarification or supplementary information. No specific decision rules were applied for selecting data from multiple reports corresponding to a single study, as each included report was treated as a distinct source for data extraction.

### 2.6. Data Items

Data were sought for both primary and secondary outcome domains as defined in the review’s objectives. All results reported within each study that were compatible with these predefined outcome domains were sought for extraction. There were no changes made to the inclusion or definition of the outcome domains, nor to their importance during the review process.

#### 2.6.1. Primary Outcomes

The primary outcomes were considered the most important for interpreting the review’s conclusions, as they directly address the core aims of evaluating pain relief, functional restoration, and objective physiological changes.

Pain Intensity: Measured using instruments such as the Visual Analogue Scale (VAS) or Numeric Pain Rating Scale (NPRS). This outcome is critical for assessing the direct effect of interventions on the primary symptom experienced by patients.

Functional Improvement: Assessed through measures like Range of Motion (ROM) or various disability indices. This reflects the patient’s ability to perform daily activities and engage in functional movements, providing a practical measure of effectiveness.

Neuromuscular Performance: Measured by relevant indicators (e.g., muscle strength, endurance, activation patterns). This outcome allows for an understanding of the physiological impact of the interventions on muscle function.

#### 2.6.2. Secondary Outcomes

Safety and Adverse Effects: Data on any reported adverse events or side effects associated with the interventions were collected to evaluate their safety profile.

Pressure Pain Threshold (PPT): An objective measure of local pain sensitivity, providing insights into the neurophysiological changes induced by the interventions.

Patient-Reported Outcomes (PROs): Broader measures reflecting the patient’s perspective on their health status, quality of life, and overall treatment experience.

#### 2.6.3. Other Variables

In addition to the outcome data, the following variables were systematically collected from each included study:

Study Characteristics: This included the specific study design (e.g., RCT, CCT), total sample size, and the duration of follow-up periods.

Population Details: Relevant demographic and clinical characteristics of the participants were extracted, such as the specific diagnosis (myofascial pain syndrome or tendinopathy), age range, and activity level.

Intervention and Comparator Protocols: Detailed information on the administered interventions (PNE, IMES) and comparators (e.g., sham, placebo, dry needling, conventional physical therapy, pharmacological treatment, no intervention) was collected. This encompassed specific parameters (e.g., intensity, frequency, duration of sessions), total treatment duration, and any concomitant therapies.

Methodological Quality Markers: Data necessary for assessing the methodological quality and risk of bias of each study were extracted, as outlined in Section 2.5.

Funding Sources: Information regarding the funding sources for each study was collected to assess potential conflicts of interest.

Assumptions regarding missing or unclear information from studies were minimized by contacting study authors for clarification. In cases where information remained unavailable after author contact, this was noted, and the impact on the synthesis was considered during the discussion of limitations. No specific external tool was used to inform which data items to collect beyond the internal standardized Excel form developed for this review.

### 2.7. Study Risk of Bias Assessment

The methodological quality and risk of bias of the included studies were independently assessed by two reviewers. For randomized controlled trials (RCTs), the Cochrane Risk of Bias tool for Randomized Trials (RoB 2) [28,29] was used. This tool assesses bias across five domains: bias arising from the randomization process, bias due to deviations from intended interventions, bias due to missing outcome data, bias in measurement of the outcome, and bias in selection of the reported result. Each domain was judged as ‘low risk of bias,’ ‘some concerns,’ or ‘high risk of bias.’ An overall risk of bias judgment for each RCT was derived based on the specific rules outlined in the RoB 2 guidance [28,29].

For non-randomized experimental and controlled studies, the Risk Of Bias In Non-randomized Studies of Interventions (ROBINS-I) tool was employed [30]. This tool evaluates bias across seven domains: confounding, selection of participants into the study, classification of interventions, deviations from intended interventions, missing data, measurement of outcomes, and selection of the reported result. Each domain was judged as ‘low risk of bias,’ ‘moderate risk of bias,’ ‘serious risk of bias,’ ‘critical risk of bias,’ or ‘no information.’ An overall risk of bias judgment for each non-randomized study was determined based on the specific rules provided within the ROBINS-I guidance.

Any disagreements between the two independent authors during the risk of bias assessment were resolved through discussion. If consensus could not be reached, a third author was consulted for final arbitration. No adaptations were made to either the RoB 2 or ROBINS-I tools. No automation tools were used for the risk of bias assessment, nor were study investigators contacted specifically for information related to risk of bias.

### 2.8. Effect Measures

For each outcome domain, the effect measures for which data were sought were consistent with the reporting in the primary studies. For continuous outcomes such as pain intensity (e.g., VAS, NPRS), functional improvement (e.g., ROM, disability indices), pressure pain threshold (PPT), and neuromuscular performance, mean differences (MD) or standardized mean differences (SMD) were collected. The choice between MD and SMD depended on the consistency of measurement scales across studies; SMD was used when different scales or units were employed for the same outcome. For dichotomous outcomes, such as the incidence of adverse effects or specific patient-reported outcomes reported as counts, risk ratios (RR) or odds ratios (OR) were collected. No synthesized results were re-expressed to a different effect measure during data collection; all reported effect measures were extracted as presented in the original studies.

### 2.9. Synthesis Methods

All studies identified as eligible after the full-text review, irrespective of their methodological quality, were considered for inclusion in the synthesis. This ensures a comprehensive overview of the available evidence. The decision to include a study in the narrative synthesis was based solely on its adherence to the predefined eligibility criteria, rather than on the specific statistical methods employed or the presence of complete data for meta-analysis. Data collected from studies were organized and prepared for presentation and synthesis. For continuous outcomes, if standard deviations were missing, they were calculated from standard errors, confidence intervals, or *p*-values where possible, using standard statistical formulas. If raw data were presented graphically, attempts were made to extract summary statistics using digital ruler software (e.g., WebPlotDigitizer, version 4.0). If data conversions were required (e.g., converting a 0–10 pain scale to a 0–100 scale for consistency in narrative comparison), these were performed and clearly noted.

Owing to substantial clinical and methodological heterogeneity across populations, interventions, comparators, and outcome measures, meta-analysis was not considered appropriate. Therefore, the included studies were summarized descriptively, grouped by intervention (PNE or IMES), condition (MPS or tendinopathy), comparator, and follow-up period. This approach allowed for a qualitative interpretation of main findings, of highlighting trends, inconsistencies, and key findings without combining numerical data in a statistical pooling. The narrative synthesis also provided context for the methodological quality and risk of bias of the included studies. Studies were carefully examined for variations in participant characteristics (e.g., age, severity of condition), intervention parameters (e.g., dose, frequency, duration), comparator types, and outcome measurement methods. Results were qualitatively reported based on these factors, allowing for the identification of patterns or potential explanations for differences in observed effects across studies.

## 3. Results

### 3.1. Study Selection

The study selection process followed the PRISMA guidelines. A total of 705 records were identified through database searches (PubMed, Scopus, and Web of Science), with no records identified from registers. Additionally, 31 records were identified through citation searching, bringing the total number of records to 736. Before screening, 82 duplicate records were removed, resulting in 623 unique records. These 623 records were then screened, leading to the exclusion of 585 records. The remaining 39 reports were sought for retrieval, and all were successfully retrieved. Concurrently, all 32 reports identified from citation searching were sought for retrieval and successfully retrieved.

**Figure 1:**
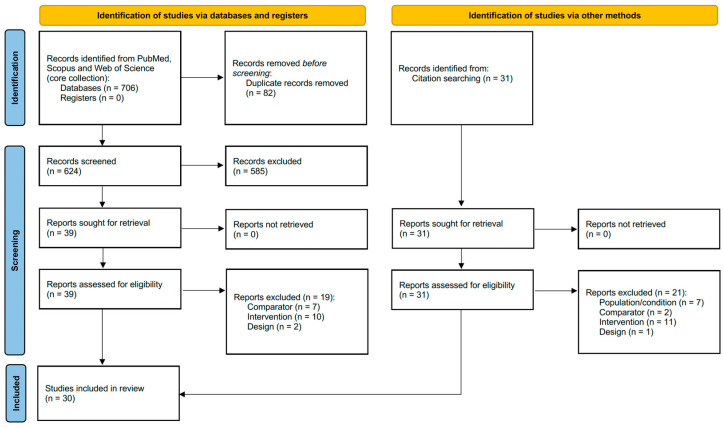
PRISMA flow diagram [27].

In the eligibility assessment phase, all 39 reports from the database searches and 32 reports from citation searching (a total of 70 reports) were assessed. From the database-identified reports, 19 were excluded for the following reasons: 7 due to comparator, 10 due to intervention, and 2 due to study design. From the citation-identified reports, 21 were excluded: 7 due to population/condition, 2 due to comparator, 11 due to intervention, and 1 due to study design. After this eligibility assessment, a total of 30 studies were deemed eligible and included in the final systematic review (Figure 1).

### 3.2. Study Characteristics

Table 2 summarizes the main methodological characteristics of the 30 included studies. The studies included a diverse group of participants, with a total sample size exceeding 1000 across the 29 compiled trials. The number of participants in each trial ranged widely, from as few as 15 [31] to 122 [32], with most studies having sample sizes between 30 and 60. The age of participants also varied, with the typical range being 18 to 65 years. For instance, the mean age in one study on myofascial pain syndrome was 50.7 ± 10.1 years [33], while a recent study on the same condition had a younger mean age of 23.33 ± 1.99 years [34]. While some studies included both males and females, several reports focused predominantly on female participants, particularly in research concerning Myofascial Pain Syndrome (MPS), with one study on the condition having 87% female participants [35] and another being 100% female [19,36].

All participants were diagnosed with a specific musculoskeletal or tendinous condition confirmed by a physician or physical therapist. The most common diagnosis was Myofascial Pain Syndrome (MPS), specifically involving the upper trapezius muscle due to the high prevalence of trigger points (TrPs) in this area [34,37,38]. Other conditions investigated included various tendinopathies, such as those of the patellar [24,39], Achilles [24], and supraspinatus tendons [25,40], as well as chronic pain in regions like the neck [38,41] and low back [32].

The IMES/PENS approach was primarily used for Myofascial Pain Syndrome and typically involved the application of a low-frequency current, commonly around 2 Hz, to trigger points within a muscle, with the intensity adjusted to evoke a painless muscle twitch [19,38]. PNE, often ultrasound-guided, was the preferred method for tendinopathies and plantar fasciopathy. It applied a galvanic current with a higher intensity, usually between 2 and 3 mA, for a short duration of a few seconds [24,42]. The frequency of treatments varied, with single-session studies being common for immediate effects [37,41], while others, focused on chronic conditions, involved multiple weekly sessions over several weeks [43,44].

The primary and secondary outcomes were selected to measure both subjective pain experience and objective physical changes. The most common primary outcome across all studies was pain intensity, which was consistently assessed using either the Numeric Pain Rating Scale (NPRS) or the Visual Analogue Scale (VAS) [34,37]. Other primary outcomes included measures of disability specific to the affected area, such as the Neck Disability Index (NDI) for neck pain [38] and the Victorian Institute of Sports Assessment–Achilles (VISA-A) for Achilles tendinopathy [24]. Secondary outcomes often focused on physical function and quality of life, including measurements of range of motion (ROM), pressure pain threshold (PPT) [41,45], and scores from validated quality of life questionnaires like the SF-12 [44] and SF-36 [39].

**Table 2:** Summary of the main methodological characteristics of the 29 included studies.

Study	Study Design	Condition	N	Age (Mean ± SD or Range)	Sex	Location of Symptoms	Intervention Group	Electrical Parameters	Number of Sessions/Treatments	Comparator	Primary Outcomes	Secondary Outcomes
Rodríguez-Huguet et al. [25]	Single-blinded, prospective and longitudinal randomized clinical trial	Supraspinatus tendinopathy	Total: 36 PE: 18 Dry Needling: 18	Range: 25–60 years	Not specified in the text	Supraspinatus tendon (shoulder)	Percutaneous electrolysis (PE) + eccentric exercise program	350 µA for 1.2 min	Weekly session for 4 weeks (4 sessions total)	Trigger point dry needling + eccentric exercise program	Pain intensity (NPRS)	Pressure pain threshold and range of motion
Lopez-Martos et al. [10]	Randomized, double-blind, single-center clinical trial	Myofascial pain syndrome (MPS) in the lateral pterygoid muscle (LPM)	Total: 60	Range: 18–65 years	Not specified in the text	Lateral pterygoid muscle (LPM)	PNE group: Percutaneous needle electrolysis DDN group: Deep dry needling	PNE: 2 mA for 3 s, three times DDN: Not applicable, but simulated noise	3 consecutive weeks (once per week)	Sham needle puncture (SNP): Needle pressed against skin with its protective tube	Pain at rest and with mastication (VAS), Maximum interincisal opening (MIO), and TMJ involvement (100-point questionnaire)	Patient and observer efficacy scores, tolerability, and adverse events
Abat et al. [42]	Randomized controlled trial (single-blind)	Patellar tendinopathy	Total: 60 USGET: 30 Electro-physiotherapy: 30	USGET: 31.2 ± 6.5 years Electro-physiotherapy: 30.9 ± 5.9 years	Male: 51 Female: 13	Patellar tendon (knee)	Ultrasound-guided galvanic electrolysis technique (USGET) + eccentric exercise	USGET: 2 mA intensity	Treatments for 2 months or until symptoms are not present (VISA-P ≥ 90)	Standard electrophysiotherapy + eccentric exercises	Symptoms, function, and ability to perform sport (VISA-P score)	Not specified in the methods section provided
Miguel-Valtierra et al. [40]	Randomized, parallel-group clinical trial	Subacromial pain syndrome	Total: 50 Each group: 25	Range: 18–65 years	Not specified in the text	Supraspinatus tendon (shoulder)	Manual therapy and exercise + US-guided percutaneous electrolysis	350 mA intensity for 90 s	Once per week for 5 weeks	Manual therapy and exercise alone	Shoulder pain related disability (DASH)	Mean, worst, and lowest shoulder pain intensity (NPRS), function (SPADI), pressure pain sensitivity (PPTs), self-perceived improvement (GROC)
Fernández-Rodríguez et al. [46]	Parallel, group-blinded, randomized, placebo-controlled trial	Chronic painful heel pain (CPHP)/Plantar fasciopathy	Total: 73 PNE: 39 Sham: 34	Mean: 45.1 ± 11.4 years	Female: 60.5%	Plantar fascia (heel)	Ultrasound-guided PNE + exercise program	28 mC (microcoulombs) of cathodal PNE	Once per week for 5 weeks	Sham intervention + exercise program	“First step” pain (NPRS)	Function (FAAM activities of daily living subscale), plantar fascia thickness, and adverse events
Rodríguez-Huguet et al. [47]	Single-blind randomized controlled clinical trial	Lateral epicondylitis (LE)	Total: 32 Each group: 16	Range: 18–60 years	Not specified in the text	Common extensor tendon (epicondyle)	Percutaneous electrolysis (PE) + eccentric exercise protocol	350 µA for 1.2 min	Once per week for 4 weeks	Trigger point dry needling (TDN) + eccentric exercise protocol	Pain intensity (NPRS)	Pressure pain threshold (PPT), elbow joint motion, and quality of life (SF-12)
Moreno et al. [48]	Single-blind randomized controlled clinical trial	Adductor-related groin pain (ALErGP)	Total: 22 Study group: 12 Control group: 10	Mean: 26.0 ± 4.7 years	100% male	Adductor longus (pubic tubercle)	Ultrasound-guided EPI^®^ + standardized active physical therapy (APT)	3 mA current intensity 5 s duration 3 applications per session	Two sessions per week during Phase 1 of APT (number of sessions depended on symptomatic improvement)	Standardized active physical therapy (APT) program alone	Pain (NRS)	Patient-Specific Functional Scale (PSFS)
Al-Boloushi et al. [43]	Prospective, parallel-group RCT with blinded outcome assessment	Chronic painful heel pain (PHP)	Total: 102 DN: 51 PNE: 51	Range: 21–60 years	Not specified in the text	Plantar and calf muscles	Percutaneous needle electrolysis (PNE) + self-stretching protocol	1.5 mA (intensity adapted to patient tolerance)	Once per week for 4 sessions	Dry needling (DN) + self-stretching protocol	Foot Pain domain (FHSQ)	Foot Function, Footwear and General Foot Health (FHSQ), average and maximum pain (VAS), and Quality of life (EQ-5D-5L)
López-Royo et al. [39]	RCT with blinded assessors and participants	Patellar tendinopathy (PT)	Total: 57 PNE: 19 DN: 19 Sham: 19	Range: 18–45 years	Not specified in the text	Inferior pole of the patella	PNE + EE (Percutaneous needle electrolysis + eccentric exercise) DN + EE (Dry needling + eccentric exercise)	PNE: 3 mA galvanic current for 3 s	4 sessions, every 2 weeks (over 8 weeks)	Sham needling + EE (sham needle on the treatment zone + eccentric exercise)	Disability (VISA-P questionnaire)	Pain level (VAS), Quality of life (SF-36), and Ultrasonographic measures (echointensity, echovariation, neovascularization)
de la Barra Ortiz et al. [49]	Randomized double-blind clinical trial	Myofascial trigger points (MTrPs) in shorter upper trapezius muscle	Total: 48 MEP: 24 Control: 24	Mean: 22 years	Male: 23 Female: 25	Shorter upper trapezius muscle	Microelectrolysis (MEP) + therapeutic ultrasound	0.6 mA at the needle	Single intervention	Therapeutic ultrasound alone	Pressure pain threshold (PPT) and Pain intensity (PI) (VAS)	Not specified in the text
Byeon et al., 2003 [33]	Randomized controlled trial (RCT)	Myofascial pain syndrome in upper trapezius muscle	Total: 30 Dry Needling: 10 IMES: 10 IMS: 10	Mean: 50.7 ± 10.1 years Range: 37–67 years	Male: 18 Female: 12	Upper trapezius muscle	Intramuscular electrical stimulation (IMES) and Intramuscular stimulation (IMS)	IMES: 10 Hz biphasic wave, 15 min, at 3× sensory threshold IMS: 10 Hz biphasic wave, 15 min, at 3× sensory threshold	3 times per week for 2 weeks	Dry needling	Pain (Visual Analog Scale) and Pain (McGill Pain Questionnaire)	Passive range of motion of cervical lateral flexion
Sumen et al. [50]	Randomized controlled trial (RCT)	Myofascial pain syndrome (MPS) in upper trapezius muscle	Total: 47 Group 1: 16 Group 2: 15 Group 3: 16	Mean: 39.0 ± 11.65 years Range: 18–65 years	Female: 32 Male: 15	Upper trapezius muscle	Intramuscular stimulation (IMS) + stretching exercises	80 Hz frequency, current increased until patient feels it	10 sessions (20 min per session)	Group 1: Stretching exercises + LLLT Group 3: Stretching exercises alone	Pain intensity (VAS), Pain threshold (PT)	Active range of motion (ROM) of the cervical spine, Neck disability index (NDI)
Medeiros et al. [36]	Randomized, double-blind, factorial design, and controlled placebo-sham clinical trial	Myofascial pain syndrome (MPS)	Total: 44	Range: 19–75 years	100% female	Not specified in the text	Deep Intramuscular Stimulation Therapy (DIMST) + rTMS	DIMST: 2 Hz frequency, 20 min duration	10 sessions	Sham-DIMST (sham electroacupuncture device) or rTMS/Sham-rTMS	Pain (VAS) and BDNF serum level	Peripheral biomarkers (S100b, LDH, TNF-a, IL-6, IL-10, SOD, catalase activity, GPx, protein carbonyls, ROS), Cortical excitability parameters (MEP, ICF, CSP, SICI)
Hadizadeh et al. [51]	Randomized double-blind clinical trial	Myofascial trigger points (MTrP) in upper trapezius muscle	Total: 16 IMES: 8 Placebo: 8	Range: 18–40 years	Not specified in the text	Upper trapezius muscle	Intramuscular electrical stimulation (IMES)	Burst frequency of 2 Hz, pulse width of 200 µs	Single session (10 min)	Dry needling alone (placebo)	Pain intensity (VAS)	Cervical spine lateral flexion Range of Motion (ROM)
Botelho et al. [19]	Randomized, double-blind, two-group parallel, clinical trial	Myofascial pain syndrome (MPS) in the upper body part	Total: 24 Each group: 12	Range: 19–65 years	100% female	Splenius capitis and semispinalis capitis (upper body)	Electrical Intramuscular Stimulation (EIMS)	2 Hz frequency, 20 min duration	10 sessions	Sham of Intramuscular Electrical Stimulation (EIMS)	Disability (Brazilian Profile of Chronic Pain: Screen) and Pain (VAS)	Analgesic doses, Numerical Pain Rating Scale (NPRS), Heat pain threshold (HPT), Sleep quality (VASQS), Cortical excitability (MEP, ICF, CSP, SICI), BDNF serum level
Brennan et al. [35]	Randomized controlled trial (RCT)	Active myofascial trigger points (MTrPs) in the upper trapezius muscle	Total: 45 DN: 25 DN/IMES: 20	DN: 26.32 ± 8.94 years DN/IMES: 28 ± 9.99 years	Male: 8 Female: 37	Upper trapezius muscle	Dry Needling (DN) with Intramuscular Electrical Stimulation (IMES)	Approx. 10 Hz frequency, intensity increased until strong but tolerable	1 treatment per week for 6 weeks	Dry Needling (DN) alone	Neck Disability Index (NDI) and Numeric Pain Rating Scale (NPRS)	Not specified in the text
Conti et al. [31]	Single-blind, randomized clinical trial	Myofascial pain syndrome (MPS) and sleep bruxism	Total: 15 Active: 7 Control: 8	Active: 37.3 ± 8.9 years Control: 31.9 ± 12.3 years Range: 20–50 years	Male: 3 Female: 12	Masseter and anterior temporalis muscles (jaw)	Transcutaneous electrical stimulation (as a feedback mechanism from EMG)	Not specified in the text, intensity was adjusted by patient to a non-painful level	At least 10 nights	Device that recorded EMG activity but did not provide stimulation	Pain intensity (VAS)	Pressure pain threshold (PPT), number of EMG events per hour (EMG/h)
Shanmugam et al. [52]	Randomized clinical trial	Myofascial trigger points (MTrPs) in the upper trapezius muscle	Total: 36 Group 1: 12 Group 2: 12 Group 3: 12	Range: 18–25 years	100% male	Upper trapezius muscle (UT)	IMES with IEP (Intramuscular Electrical Stimulation with Injured Electrode Polarity) and IMES with CEP (Intramuscular Electrical Stimulation with Conventional Electrode Polarity)	5 Hz frequency, 500-µs pulse duration, intensity tolerable by participants (0–140 mA)	Single session (10 min)	Sham-IMES (very minimal intensity)	Pressure pain threshold (PPT) and Upper trapezius (UT) muscle activity (EMG RMS)	Pain severity (VAS) and UT muscle length
Hadizadeh et al. [37]	Double-blind, randomized controlled trial	Myofascial trigger points (MTrPs) in upper trapezius muscle	Total: 30 IMES: 15 Placebo: 15	Range: 18–40 years	Not specified in the text	Upper trapezius muscle	Intramuscular electrical stimulation (IMES)	Burst frequency of 2 Hz, pulse duration of 200 µs, intensity increased until painless contractions created	Single session (10 min)	Dry needling with sham electrical current (placebo)	Pain (VAS) and disability (NDI)	Pressure pain threshold (PPT) and cervical lateral flexion ROM
León-Hernández et al. [38]	Single-blinded randomized controlled trial	Chronic non-specific neck pain	Total: 62 DN: 31 DN + PENS: 31	Mean: 25 ± 8 years Range: 18–48 years	Male: 16 Female: 46	Upper trapezius muscle	Dry Needling (DN) with Percutaneous Electrical Nerve Stimulation (PENS)	2 Hz frequency, 120 µs pulse width, intensity to be well tolerated and not painful	Single treatment (15 min)	Dry Needling (DN) alone	Post-needling soreness (VAS), Neck pain intensity (VAS), and Disability (NDI)	Pressure pain threshold (PPT) and Cervical Range of Motion (CROM)
Garcia-de-Miguel et al. [41]	Randomized controlled trial (RCT)	Non-traumatic unilateral mechanical neck pain with active MTrP in levator scapulae	Total: 44 DN: 22 PENS: 22	Range: 18+ years	Not specified in the text	Levator scapulae muscle	Percutaneous Electrical Nerve Stimulation (PENS) + dry needling	2 Hz frequency, 100 µs pulse width, intensity to patient’s tolerance (max 3 mA)	Single treatment (20 min)	Dry Needling (DN) alone	Pain intensity (VAS), pressure pain threshold (PPT), side-bending strength, and range of movement	Disability (NDI)
Pérez-Palomares et al. [32]	Pragmatic clinical trial	Nonspecific chronic low back pain (CLBP)	Total: 122 PENS: 67 DN: 68	Mean: 45.85 ± 14.4 years	Male: 31 Female: 91	Deep lumbar paraspinal muscles, quadratus lumborum, and gluteus medius	Percutaneous Electrical Nerve Stimulation (PENS)	4 Hz frequency, 0.3 ms pulse duration	9 sessions over 3 weeks	Dry Needling (DN)	Perceived pain (VAS), pain tolerance (PPT), sleep quality (VAS), and quality of life (Oswestry Disability Index)	Not specified in the methods section provided
Niddam et al. [53]	Randomized controlled trial (RCT) using fMRI	Myofascial pain syndrome (MPS)	Total: 24 G1: 12 G2: 12	Not specified in the text	Not specified in the text	Upper left trapezius muscle	Intramuscular electrical stimulation (IMES)	2 Hz frequency, 1 ms pulse, intensity to be “intense but nonpainful” and induce visible muscle contraction	Single intervention (3 min)	Not applicable, as it was a within-subject, between-session comparison with a sham-like protocol	Psychophysical pain threshold (PT) and Pressure pain threshold (PPT)	Not applicable, as the primary outcomes are psychophysical, not clinical. The main purpose was to analyze brain activity.
Di Gesù et al. [24]	Randomized controlled pilot study (RCT), single-blind	Unilateral non-insertional Achilles tendinopathy (AT)	Total: 50 EG: 25 CG: 25	Mean: 40.92 ± 9.70 years Range: 25–60 years	Male: 32 (64%) Female: 18 (36%)	Achilles tendon	High-intensity ultrasound-guided galvanic electrolysis technique (HI-USGET) + eccentric exercises	2 mA intensity for 10 s; 100 µs pulse width; 100 Hz frequency	3 treatments (15 days apart) + 8 weeks of exercises	Eccentric exercises alone	Severity of condition (VISA-A)	Pain intensity (VAS)
Doménech-García et al. [54]	Randomized double-blinded controlled trial (RCT), three-arm	Anterior knee pain/Patellar tendinopathy	Total: 42 Each group: 14	Range: 18–45 years	Not specified in the text	Patellar tendon (knee)	Percutaneous Needle Electrolysis (PNE) + eccentric exercise program	3 mA galvanic current for 3 s per insertion (3 insertions)	4 sessions over 8 weeks + daily eccentric exercises	Dry needling (with 0 mA current) + exercises and Sham needle + exercises	Clinical pain reduction after intervention and needle-related pain intensity	Clinical pain intensity, needle-related pain during intervention, time until pain disappears, VISA-p, and ultrasonographic measures
Ga et al. [55]	Randomized controlled trial (RCT)	Chronic myofascial pain syndrome (MPS)	Total: 43 IMS: 21 TPI: 22	Not specified in the provided text	Male: 5 Female: 38	Upper trapezius muscle	Intramuscular Stimulation (IMS) + self-stretching exercises	“Grasping and winding up” nerve root stimulation at C3–5 level (specific electrical parameters not mentioned in the text)	Treatments at weeks 0, 1, and 2 + daily self-stretching exercises	Trigger Point Injection (TPI) with 0.5% lidocaine + self-stretching exercises	Pain intensity (VAS, Wong-Baker FACES) and Pain pressure threshold scores (PTS)	Passive range of motion (ROM) and Depression (GDS-SF)
Hadizadeh et al. [45]	Randomized, single-blind clinical trial (RCT)	Chronic myofascial pain syndrome (MPS) with active trigger points (TrPs)	Total: 30 IMES: 15 DN: 15	Range: 18–50 years	Male: 6 Female: 24	Upper trapezius (UT) muscle	Intramuscular Electrical Stimulation (IMES)	Burst current, 2 Hz frequency, 200 µs pulse width, 10 min duration. Intensity increased until painless contraction.	3 sessions during one week	Dry Needling (DN)	Cervical lateral flexion ROM and TrP circumference (CIR)	Pain intensity (VAS), pain pressure threshold (PPT), NDI, TrP diameters, TrP stiffness, and blood circulation
Zuccolotto Moro et al. [44]	Prospective, randomized, open-label clinical trial	Myofascial pain syndrome (MPS)	Total: 90 (30 per group)	Range: > 18 years	Not specified	Upper trapezius muscle (neck and shoulders)	Group 2: TrP needling with electrical stimulation	Direct current, 2 Hz frequency, 200 ms pulse duration, 3–4 mA intensity	7 weekly sessions	Group 1: Dry needling (DN) of TrPs<br>Group 3: Motor point/nerve needling with electrical stimulation	Pain score (VAS)	Health-related quality of life (SF-12)
Sharma et al. [34]	Randomized clinical trial (RCT)	Active myofascial trigger points (TrPs)	Total: 45 DN: 15 DN/IMES [2 Hz]: 15 DN/IMES [100 Hz]: 15	Mean: 23.33 ± 1.99 years Range: 18–30 years	Male: 23 Female: 22	Upper trapezius muscle	Dry Needling (DN) with Intramuscular Electrical Stimulation (IMES)	Biphasic current, 200 µs pulse width, at two different frequencies: 2 Hz and 100 Hz	Single intervention (10 min)	Dry Needling (DN) alone	Numeric pain rating scale (NPRS) and Pain pressure threshold (PPT)	Not specified in the text
Rejas-Fernández et al. [56]	Randomized clinical trial	Tibialis posterior tendinopathy	Total: 46 percutaneous electrolysis + manual therapy/exercise: 23 MT/exercise: 23	Adults	Not specified	Tibialis posterior tendon/medial ankle-foot region	Ultrasound-guided percutaneous electrolysis + manual therapy and exercise program	Not specified	1 session/week for 4 weeks (4 sessions total); PE applied at each treatment session	Manual therapy and exercise program alone	Pain intensity (Numerical Pain Rating Scale, NPRS)	Disability (Foot and Ankle Ability Measure, FAAM-ADL and FAAM-Sports)

### 3.3. Risk of Bias in Studies

The overall risk of bias across the included studies was deemed high, with 16 out of the 30 studies rated as having an overall high risk of bias (Table 3). This was primarily driven by issues related to deviations from intended interventions (n = 14) and missing outcome data (n = 5). This was largely because it was impossible to blind participants and personnel to the interventions, which involved physical treatments such as electrical stimulation. This creates a substantial potential for performance bias, as both patient expectations and practitioner behavior could influence the outcomes.

Furthermore, this lack of blinding frequently led to a high risk of detection bias when subjective outcomes were measured by unblinded assessors, with 5 studies rated as having a high risk in this domain. While some studies successfully mitigated this risk for objective outcomes (e.g., imaging), the unblinded assessment of subjective measures remains a critical concern.

### 3.4. Results of Individual Studies

#### 3.4.1. Percutaneous Needle Electrolysis (PNE)

PNE, including variants such as ultrasound-guided galvanic electrolysis technique (USGET) and percutaneous microelectrolysis (MEP), generally showed favorable effects on pain intensity across several studies (Table 4, Table 5 and Table 6), with significant between-group improvements reported in supraspinatus tendinopathy [33], temporomandibular myofascial pain [10], subacromial pain syndrome [34], plantar fasciopathy [40], lateral epicondylitis [41], adductor-related groin pain [42], and, at least at short-term follow-up, Achilles tendinopathy [31]. However, superiority over comparators was not consistent across all conditions. No significant between-group differences in pain reduction were observed in chronic painful heel pain compared with dry needling [37], patellar tendinopathy when PNE was combined with eccentric exercise [39], myofascial trigger point-related pain compared with therapeutic ultrasound [43], and patellar tendinopathy in the study by Doménech-García et al. [54], in which clinical pain decreased in all groups without significant differences between them.

In terms of functional improvement, PNE was frequently associated with significant improvements in Range of Motion (ROM) [25,47]. However, PNE was not significantly better than comparators in some disability measures like the DASH [40] or most sections of the Oswestry Disability Index [32]. PNE also showed positive effects in Pressure Pain Threshold (PPT) [25,47,49]. Some studies also noted PNE’s superior effect on patient-reported and observer-reported efficacy [10] and global rating of change [40].

#### 3.4.2. Intramuscular Electrical Stimulation (IMES)

IMES generally showed effectiveness in improving pain and function, though with some inconsistencies (Table 7). IMES was often effective in reducing pain intensity [19,33,51,52]. In terms of functional improvement, IMES consistently showed benefits in cervical ROM [33,37,51]. However, IMES was not significantly different from comparators in neck disability in several studies [35,38,50].

Regarding neuromuscular performance and other outcomes, IMES was consistently found to increase PPT [38,52,53]. However, it was not significantly better than comparators in quality of life measures like SF-12 [44].

#### 3.4.3. Safety and Adverse Effects

Many studies explicitly stated that no adverse events were reported in association with the interventions [42,46,49,54]. However, minor common adverse events noted in PNE and IMES included: Muscle soreness, reported in PNE [40] and IMES [55], typically resolving spontaneously within 24–36 h; hematomas, as small, self-limiting hematomas were occasionally observed in both PNE [10,43] and IMES [55]; vasovagal responses, experienced by a few participants in one study combining dry needling with IMES [35].

## 4. Discussion

This systematic review evaluated the effects of percutaneous needle electrolysis (PNE) and intramuscular electrical stimulation (IMES) in adults with myofascial pain syndrome and tendinopathies. Overall, the included studies suggest that these interventions may improve pain and selected functional or neurophysiological outcomes. However, these findings should be interpreted cautiously because the evidence base was heterogeneous, frequently at high risk of bias, and several studies assessed PNE or IMES as part of a combined intervention with exercise, manual therapy, or dry needling. In such cases, the observed effects should be interpreted as the added value of PNE or IMES within a broader rehabilitation strategy, rather than as the isolated effect of the electrical needling technique itself. Reported adverse events were generally mild and transient.

### 4.1. Percutaneous Needle Electrolysis (PNE)

The evidence reported in this review suggests that PNE may be beneficial in some myofascial pain and tendinopathy presentations, particularly for pain reduction. However, this interpretation should be tempered by the fact that several PNE studies evaluated the technique as an adjunct to exercise or manual therapy rather than as a stand-alone intervention [24,25,40,46,47]. Therefore, in studies such as those on supraspinatus tendinopathy, Achilles tendinopathy, subacromial pain syndrome, and some patellar tendinopathy protocols, the findings reflect the effect of adding PNE to an active rehabilitation program or comparing it with another active intervention, rather than a pure estimate of PNE alone. However, this analgesic effect was not universally superior. Some studies reported that PNE was comparable to other interventions in certain contexts, including chronic painful heel pain when compared with dry needling [37], patellar tendinopathy when combined with eccentric exercise [39], and myofascial trigger point-related pain when compared with therapeutic ultrasound [43]. Importantly, Doménech-García et al. [54] also found that PNE was not significantly better than sham comparators in clinical pain reduction in patellar tendinopathy, with pain decreasing similarly across all groups.

Regarding functional outcomes, the evidence is more convincing for some specific between-group comparisons than for a general functional superiority of PNE [25,47]. Clear between-group advantages in favor of PNE were reported for range of motion in studies involving shoulder and elbow-related conditions, for foot and ankle function measured with the FAAM-ADL in plantar fasciopathy [46], and for Achilles tendinopathy severity measured with the VISA-A when PNE was added to eccentric exercise [24]. Between-group benefits were also observed in some patient-reported outcomes, such as patient/observer-rated efficacy [10] and global rating of change [40]. In contrast, other outcomes showed either only within-group improvement or no statistically significant between-group superiority, including quality-of-life improvement in the PNE plus eccentric exercise arm of patellar tendinopathy, shoulder disability measured with the DASH in subacromial pain syndrome [40], and most disability domains in low back pain-related studies. Therefore, the functional effects of PNE should be interpreted as outcome-specific and condition-dependent, rather than uniformly superior across all comparators.

From a neuromuscular and physiological perspective, PNE consistently showed a significant ability to increase PPT, indicating a reduction in local tissue sensitivity, often surpassing control or comparator interventions [25,47,49]. This finding suggests a localized neurophysiological effect that contributes to its therapeutic benefits. However, some studies reported no significant differences in PPT at certain anatomical locations [32,40] or in structural changes within tendons [39], indicating that its mechanisms of action might be more complex than solely structural tissue repair or broad pain threshold modulation.

Crucially, the safety profile of PNE appears favorable. Across the included studies, reported adverse events were consistently limited, predominantly minor, and self-limiting. These typically included transient muscle soreness [40] and occasional small, self-resolving hematomas [10,43]. A major number of studies explicitly stated the absence of any adverse events related to the intervention [24,42,46,49,54]. Comparative analyses generally found PNE to be not significantly different from control or comparator groups regarding adverse events, suggesting a similar safety profile to other percutaneous techniques or conservative treatments. This robust safety record is a critical consideration for its clinical application.

### 4.2. Intramuscular Electrical Stimulation (IMES)

The available evidence suggests that IMES may reduce pain in selected myofascial pain-related conditions, particularly when compared with sham or placebo procedures [19,33,51,52]. However, this interpretation also requires caution because some studies assessed IMES as an adjunct to dry needling rather than as an isolated intervention [35,41,45]. In those designs, the findings should be interpreted as the additional effect of electrical stimulation over and above the accompanying needling intervention. Moreover, the analgesic effects of IMES were not uniform across all studies, as some trials reported no clear superiority over dry needling or low-level laser therapy [50].

In terms of functional improvement, IMES frequently led to significant gains in cervical ROM [33,37,51], indicating its role in restoring mobility. It also showed effectiveness in reducing disability linked to pain [19] and improving muscle length [52]. However, the benefits in functional outcomes were not universally observed, with several studies finding no significant difference between IMES and comparators in overall neck disability [35,38,50] or showing mixed results for various ROM measures [38,41].

From a neurophysiological perspective, several studies [38,52,53] reported comparator-based improvements after IMES, but these findings were not uniform across all outcomes or studies. Increases in pressure pain threshold were demonstrated in some sham-, placebo-, or dry needling-controlled studies, suggesting a possible desensitization effect in selected myofascial pain presentations. However, this effect was not observed consistently across all comparators, as some studies did not find significant between-group differences in pressure pain threshold. Other neurophysiological findings should be interpreted more cautiously. Modulation of periaqueductal gray activity was reported in a responder-based analysis [53] rather than in a straightforward between-group design, reductions in electromyographic events during sleep were observed in one bruxism study [31] with partial comparator superiority, and improvements in sleep quality, cortical excitability markers, and serum BDNF [19] were reported mainly in single sham-controlled trials. Therefore, while IMES may influence neurophysiological parameters, the current evidence remains preliminary and does not yet support a consistent conclusion of superiority across all comparator conditions.

The safety profile of IMES is an important consideration for its clinical application. Consistent with PNE, reported adverse events associated with IMES were generally limited, mild, and transient. These typically included self-limiting muscle soreness [55]. Some studies explicitly reported no adverse events [19,36]. Comparative analyses across the included studies found IMES to be not significantly different from control or comparator groups regarding the occurrence of adverse events [19,31,35,36,52]. This consistent finding underlines the generally safe nature of IMES, reinforcing its viability as a therapeutic option.

### 4.3. Study Limitations and Future Research

A critical limitation across the body of evidence reviewed is the inherent difficulty in blinding participants and practitioners to the interventions. The physical nature of procedures like PNE and IMES makes it nearly impossible to implement a truly double-blind design, which introduces a substantial risk of performance and detection bias. This limitation, which was a concern in some included studies, may lead to inflated perceptions of treatment efficacy, as patient expectations and practitioner-patient interactions could influence subjective outcomes such as pain ratings. Future research must address this by prioritizing the development and validation of more credible sham procedures that can genuinely mimic the sensation of active treatment without delivering the therapeutic effect.

Moreover, while objective measures were used in some studies, future research should expand the use of such outcomes—including those beyond imaging, such as biomechanical or neurophysiological data—that are less susceptible to bias. A significant gap in the current literature is the lack of standardized, objective outcome measures that can reliably capture the effects of these interventions, independent of patient and assessor expectations. Therefore, a key recommendation for future studies is to integrate a mixed-methods approach that combines traditional subjective scales with a battery of objective, quantifiable measures. This would provide a more robust basis for evaluating the true therapeutic effects of these interventions, independent of placebo effects, and would enhance the overall quality and trustworthiness of the evidence.

A third limitation and source of heterogeneity is the wide variation in the technical application of PNE and IMES across studies. There is no clear consensus on optimal treatment parameters, with significant differences in current intensity (e.g., 2 mA vs. 3 mA), frequency (e.g., 2 Hz vs. 100 Hz), and the duration of stimulation. Furthermore, the number of treatment sessions and the intervals between them (e.g., 3 sessions in one week vs. 7 weekly sessions) vary substantially, making direct comparisons between studies difficult. This lack of standardization extends to the specific treatment procedures, such as the use of an electrical stimulator vs. a needle with a plunger, and whether the intervention targets a trigger point, a nerve, or a motor point. These methodological inconsistencies contribute to the overall heterogeneity of the findings and make it challenging to synthesize the results and draw firm conclusions about the most effective treatment protocol.

A further important limitation of this review is the lack of homogeneity in the anatomical target structures treated. Across the included studies, PNE and IMES were applied to different tissues, including myofascial trigger points, muscle, tendons, and peritendinous structures, often across distinct clinical conditions. This makes the interpretation of pooled narrative findings more difficult, because improvements in outcomes such as range of motion or pain sensitivity may reflect different mechanisms depending on the target tissue and pathology involved. Consequently, the generalizability of the results is limited. Future research should standardize and report more clearly the anatomical target, pathological condition, and needling rationale, and should stratify analyses according to the structure treated.

### 4.4. Practical Applications

The available evidence suggests that PNE may be considered as an adjunct to exercise-based rehabilitation in tendinopathies, particularly when pain and local mechanical sensitivity remain persistent despite conventional care. Its use appears most justifiable when combined with an active rehabilitation program and when the co-intervention is standardized across treatment approaches. However, the current evidence does not support assuming superiority over all other conservative strategies, and PNE should be applied as part of a multimodal plan rather than as a stand-alone solution.

In patients with MPS or trigger point-related muscle pain, PNE may be considered when the clinical presentation is dominated by localized pain, reduced pressure pain threshold, or movement restriction associated with a defined symptomatic tissue target. Nevertheless, the evidence for PNE in myofascial pain is less consistent than in tendinopathy-related conditions, and its comparative advantage over other needling-based approaches, such as dry needling, remains uncertain in some contexts.

IMES may be considered in selected patients with myofascial pain syndrome, particularly when the treatment goal includes pain modulation, improvement in pressure pain threshold, or recovery of cervical range of motion. The intervention may be most clinically relevant in patients with clear trigger point-related symptoms and when administered by clinicians experienced in needle-based electrotherapy. However, substantial variability in stimulation parameters, treatment dosage, and anatomical targets limits the ability to define an optimal protocol or to generalize results across patient groups. At present, the evidence is insufficient to support routine clinical use of IMES in tendinopathies. Compared with PNE, the tendinopathy-specific literature for IMES is limited and does not allow conclusions regarding its effectiveness, appropriate indications, or comparative value within tendon rehabilitation.

Across both interventions, the reported adverse effects were generally mild and transient, most commonly post-treatment soreness or small self-limiting local reactions. Given the heterogeneity of the included studies and the frequent high risk of bias, the following practical implications should be interpreted as cautious clinical considerations rather than firm recommendations.

## 5. Conclusions

This systematic review suggests that percutaneous needle electrolysis (PNE) and intramuscular electrical stimulation (IMES) may reduce pain and improve selected functional outcomes in adults with myofascial pain syndrome and tendinopathies. PNE showed more consistent benefits across tendinopathy studies, whereas IMES appeared more interesting for pain reduction and some neuromuscular outcomes, such as cervical range of motion and pressure pain threshold, in myofascial pain-related conditions. However, these findings should be interpreted cautiously because the included studies were heterogeneous and frequently at high risk of bias, with half of studies rated as overall high risk, although reported adverse events were generally mild and transient.

## Figures and Tables

**Table 1:** Search strategy used for PubMed, Scopus, and Web of Science Core Collection.

Database	Field Model	Search String
PubMed	Title/Abstract	(“percutaneous needle electrolysis”[Title/Abstract] OR “galvanic electrolysis”[Title/Abstract] OR “percutaneous electrolysis”[Title/Abstract] OR “needle electrolysis”[Title/Abstract] OR “PNE”[Title/Abstract] OR “electrolysis”[Title/Abstract] OR “intramuscular electrical stimulation”[Title/Abstract] OR “IMES”[Title/Abstract] OR “EIMS”[Title/Abstract] OR “electrical intramuscular stimulation”[Title/Abstract] OR “intramuscular stimulation”[Title/Abstract] OR “electrotherapy”[Title/Abstract] OR “electroacupuncture”[Title/Abstract]) AND (“myofascial pain syndrome”[Title/Abstract] OR “myofascial pain”[Title/Abstract] OR “trigger point”[Title/Abstract] OR “muscle pain”[Title/Abstract] OR “tendinopathy”[Title/Abstract] OR “tendinopathies”[Title/Abstract])
Scopus	Title/Abstract	TITLE-ABS(“percutaneous needle electrolysis” OR “galvanic electrolysis” OR “percutaneous electrolysis” OR “needle electrolysis” OR “PNE” OR “electrolysis” OR “intramuscular electrical stimulation” OR “IMES” OR “EIMS” OR “electrical intramuscular stimulation” OR “intramuscular stimulation” OR “electrotherapy” OR “electroacupuncture”) AND TITLE-ABS(“myofascial pain syndrome” OR “myofascial pain” OR “trigger point” OR “muscle pain” OR “tendinopathy” OR “tendinopathies”)
Web of Science Core Collection	Topic	((TI=(“percutaneous needle electrolysis” OR “galvanic electrolysis” OR “percutaneous electrolysis” OR “needle electrolysis” OR “PNE” OR “electrolysis” OR “intramuscular electrical stimulation” OR “IMES” OR “EIMS” OR “electrical intramuscular stimulation” OR “intramuscular stimulation” OR “electrotherapy” OR “electroacupuncture”)) OR (AB=(“percutaneous needle electrolysis” OR “galvanic electrolysis” OR “percutaneous electrolysis” OR “needle electrolysis” OR “PNE” OR “electrolysis” OR “intramuscular electrical stimulation” OR “IMES” OR “EIMS” OR “electrical intramuscular stimulation” OR “intramuscular stimulation” OR “electrotherapy” OR “electroacupuncture”))) AND ((TI=(“myofascial pain syndrome” OR “myofascial pain” OR “trigger point” OR “muscle pain” OR “tendinopathy” OR “tendinopathies”)) OR (AB=(“myofascial pain syndrome” OR “myofascial pain” OR “trigger point” OR “muscle pain” OR “tendinopathy” OR “tendinopathies”)))

**Table 3:** Summary of the risk of bias using RoB 2.

Study	Random Sequence Generation	Deviations from the Intended Interventions	Missing Outcome Data	Measurement of the Outcome	Selection of the Reported Result	Overall Bias
Rodríguez-Huguet et al. [25]	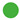	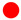	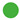	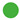	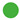	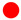
Lopez-Martos et al. [10]	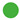	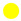	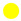	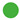	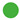	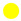
Abat et al. [42]	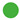	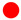	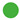	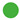	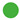	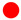
Miguel-Valtierra et al. [40]	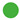	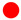	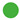	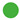	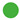	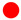
Fernández-Rodríguez et al. [46]	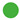	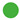	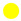	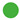	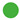	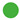
Rodríguez-Huguet et al. [47]	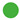	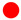	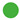	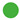	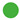	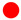
Rejas-Fernández et al. [56]	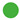	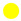	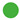	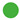	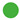	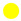
Moreno et al. [48]	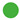	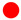	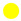	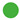	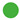	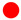
Al-Boloushi et al. [43]	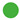	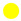	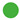	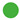	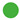	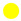
López-Royo et al. [39]	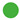	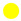	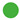	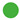	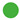	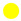
de la Barra Ortiz et al. [49]	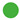	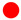	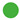	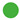	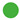	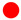
Byeon et al. [33]	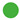	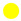	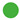	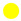	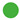	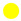
Sumen et al. [50]	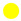	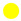	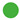	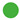	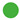	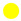
Medeiros et al. [36]	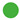	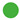	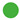	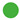	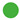	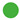
Hadizadeh et al. [51]	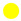	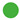	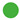	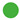	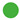	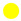
Botelho et al. [19]	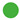	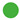	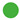	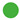	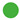	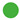
Brennan et al. [35]	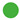	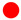	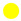	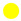	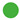	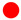
Conti et al. [31]	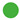	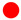	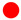	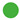	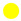	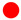
Shanmugam et al. [52]	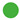	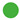	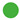	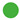	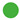	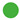
Hadizadeh et al. [37]	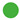	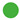	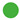	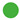	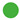	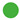
León-Hernández et al. [38]	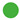	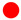	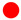	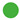	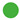	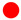
Garcia-de-Miguel et al. [41]	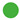	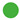	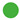	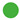	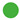	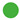
Pérez-Palomares et al. [32]	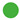	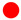	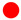	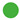	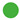	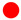
Niddam et al. [53]	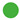	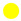	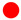	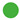	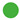	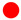
Di Gesù et al. [24]	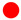	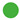	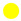	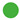	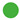	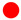
Doménech-García et al. [54]	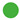	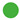	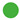	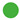	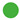	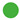
Ga et al. [55]	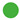	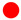	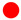	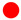	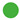	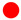
Hadizadeh et al. [45]	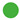	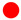	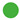	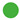	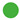	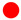
Zuccolotto Moro et al. [44]	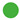	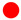	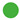	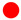	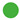	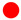
Sharma et al. [34]	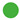	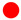	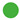	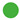	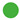	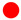

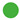
: low risk; 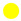: some concerns; 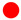:high risk.

**Table 4:** Summary of the main findings for Percutaneous Needle Electrolysis (PNE) in myofascial pain.

Study	Main Findings from Primary Outcomes	Main Findings From Secondary Outcomes
Lopez-Martos et al. [10]	Pain Intensity (Pain at rest): PNE significantly better than Sham Needling Procedure (SNP) at Day 28, Day 42, and Day 70. Deep Dry Needling (DDN) significantly better than SNP at Day 42 and Day 70. PNE significantly better than DDN at Day 28 and Day 42. Pain Intensity (Pain on mastication): PNE significantly better than SNP at Day 28, Day 42, and Day 70. DDN significantly better than SNP at Day 42 and Day 70. PNE was not significantly better than DDN in pain on mastication at any measured time point. Functional Improvement (MIO): PNE significantly better than DDN and SNP in Maximum Interincisal Opening (MIO) at all measured time points.	Patient-Reported Outcomes (Efficacy): PNE significantly better than SNP in patient-reported efficacy at all time points. PNE significantly better than DDN in patient-reported efficacy at Day 70. PNE significantly better than DDN and SNP in observer-reported efficacy. Safety/Adverse Effects: One self-limiting hematoma was reported in the PNE group. No significant differences were found in treatment tolerance between PNE, DDN, and SNP.
de la Barra Ortiz et al. [49]	Pain Intensity (PI): Percutaneous Microelectrolysis (MEP), a form of PNE, significantly decreased Pain Intensity (PI) in the experimental group at all re-evaluation sessions (Day 1: *p* = 0.0001; Day 3: *p* = 0.0001; Day 7: *p* = 0.0008). MEP was not significantly better than therapeutic ultrasound (control group) in decreasing pain intensity (PI) at any measured time point (Day 1: *p* = 0.3557; Day 3: *p* = 0.2055; Day 7: *p* = 0.2457).	Pressure Pain Threshold (PPT): MEP significantly increased Pressure Pain Threshold (PPT) in the experimental group at all re-evaluation sessions (Day 1: *p* = 0.0000; Day 3: *p* = 0.0000; Day 7: *p* = 0.0000). MEP was significantly better than therapeutic ultrasound (control group) in increasing Pressure Pain Threshold (PPT) at the second re-evaluation session (Day 3: *p* = 0.0032). MEP was not significantly better than therapeutic ultrasound (control group) in increasing PPT at Day 1 (*p* = 0.0520) or Day 7 (*p* = 0.0548). Safety/Adverse Effects: No adverse events were reported in association with the trial intervention. MEP was not reported as significantly different from therapeutic ultrasound (control group) regarding the occurrence of adverse events.
Garcia-de-Miguel et al. [41]	Pain Intensity (VAS): Percutaneous Electrical Nerve Stimulation (PENS), a form of IMES, was not significantly better than Dry Needling (DN) in pain intensity (Visual Analogue Scale—VAS) at any measured time point (*p* = 0.67 for time-by-group interaction). Functional Improvement (ROM): Mixed results. IMES (PENS) showed significantly greater improvement in cervical flexion ROM immediately post-treatment (*p* = 0.046 for time-by-group interaction, with PENS showing 7.29 mean difference vs. DN). No consistent significant differences for other ROM measures (extension, rotation, side-bending) between groups across all time points. IMES (PENS) was significantly better than DN in cervical flexion ROM immediately post-treatment but not consistently across all ROM measures or time points. Neuromuscular Performance (Side-Bending Strength): IMES (PENS) was not significantly better than DN in side-bending strength (*p* = 0.65 for time-by-group interaction).	Disability (NDI): IMES (PENS) showed significantly greater improvements in Neck Disability Index (NDI) compared to DN (mean difference, 3.27; 95% CI, 0.27–6.27; *p* = 0.03 for time-by-group interaction). IMES (PENS) was significantly better than DN in disability (NDI). Pressure Pain Threshold (PPT): IMES (PENS) showed significantly greater improvements in Pressure Pain Threshold (PPT) compared to DN (mean difference, 0.88–1.35; *p* < 0.01 for time-by-group interaction). IMES (PENS) was significantly better than DN in Pressure Pain Threshold (PPT). Safety/Adverse Effects: No adverse events were reported in the provided text. IMES (PENS) was not reported as significantly different from DN regarding safety or adverse effects.

**Table 5:** Summary of the main findings for Percutaneous Needle Electrolysis (PNE) in tendinopathy.

Study	Main Findings from Primary Outcomes	Main Findings from Secondary Outcomes
Rodríguez-Huguet et al. [25]	Pain Intensity (NPRS): Percutaneous Electrolysis (PNE) significantly better than Trigger Point Dry Needling (TDN) in pain intensity (Numerical Pain Rating Scale—NPRS) at all time points (post-treatment, 1-month, 1-year), with long-term significance (*p* = 0.002). Functional Improvement (ROM): PNE significantly better than TDN in Range of Motion (ROM) for Abduction, Extension, Internal Rotation, and External Rotation at all time points, and for Flexion at 1-month and 1-year. PNE was not significantly better than TDN for cervical flexion at the end of treatment.	Pressure Pain Threshold (PPT): PNE significantly better than TDN in proximal, middle, and distal Pressure Pain Threshold (PPT) at all time points (post-treatment, 1-month, 1-year). Safety/Adverse Effects: No significant comparative differences in safety or adverse effects were explicitly reported.
López-Royo et al. [39]	Pain Intensity (VAS): PNE combined with eccentric exercise (EE) showed significant within-group improvements in Visual Analogue Scale (VAS) pain at 10 weeks (*p* = 0.02) and 22 weeks (*p* < 0.05). PNE (combined with EE) was not significantly better than Dry Needling (DN) (combined with EE) or the control group (EE with sham needle) in mean or maximum VAS pain. Functional Improvement (VISA-p): PNE combined with EE showed significant within-group improvements in disability (Victorian Institute of Sports Assessment Questionnaire, patellar tendon—VISA-p) at 10 weeks and 22 weeks (*p* < 0.01). PNE (combined with EE) was not significantly better than DN (combined with EE) or the control group (EE with sham needle) in disability (VISA-p).	Quality of Life (SF-36): PNE combined with EE showed significant within-group improvement in Quality of Life (Short Form-36—SF-36) at 10 weeks (*p* = 0.01), but this was not maintained at 22 weeks. PNE (combined with EE) was not significantly better than DN (combined with EE) or the control group (EE with sham needle) in quality of life (SF-36). Structural Abnormalities (Ultrasound): No statistically significant changes were observed in tendon structure parameters (thickness, neovascularization, echointensity, echovariation, total area of vessels, mean area or vessel) over time or between groups. PNE (combined with EE) was not significantly different from DN (combined with EE) or the control group (EE with sham needle) in structural abnormalities (ultrasound parameters). Safety/Adverse Effects: Not explicitly reported, but no mention of adverse events in the provided text. PNE (combined with EE) was not reported as significantly different from DN (combined with EE) or the control group (EE with sham needle) regarding the occurrence of adverse events.
Di Gesù et al. [24]	Pain Intensity (VAS): Ultrasound-guided Galvanic Electrolysis Technique (USGET), a form of PNE, combined with eccentric exercise showed significantly greater pain reduction (Visual Analogue Scale—VAS) in the experimental group compared to the control group at T2 (1 month follow-up) (*p* = 0.002). At T3 (2 months follow-up), both groups improved in VAS, but the difference between groups was not statistically significant (*p* = 0.404). PNE (USGET) was significantly better than conventional eccentric exercise in pain intensity (VAS) at T2. Functional Improvement (VISA-A): PNE (USGET) combined with eccentric exercise showed significantly greater improvement in Achilles tendinopathy severity (Victorian Institute of Sport Assessment-Achilles—VISA-A) at T2 (*p* = 0.010) and T3 (*p* < 0.001) compared to conventional eccentric exercise. Specifically, at T3, the experimental group showed greater improvement across VISA-A subscales: Pain (*p* = 0.004), Function (*p* = 0.003), and Sport (*p* = 0.002). PNE (USGET) was significantly better than conventional eccentric exercise in VISA-A and its subscales (Pain, Function, Sport) at T2 and T3.	Safety/Adverse Effects: No adverse events were reported during the study and treatment with HI-USGET. PNE (USGET) was not reported as significantly different from conventional eccentric exercise regarding the occurrence of adverse events.
Doménech-García et al. [54]	Pain Intensity (Clinical pain reduction): Percutaneous needle electrolysis (PNE) was not significantly better than sham PNE groups (dry needling or sham needles) in terms of clinical pain reduction (*p* = 0.424). Clinical pain decreased in all groups (*p* < 0.001).	Needle-related pain during needle intervention: The double-sham group showed a lower percentage of participants reporting needle-related pain during needle intervention (*p* = 0.005). Needle-related pain intensity after needle intervention: Needle-related pain intensity after needle intervention was similar between groups (*p* = 0.682). PNE was not significantly different from sham PNE groups in needle-related pain intensity after needle intervention. Safety/Adverse Effects: No adverse events were reported other than the needle-related pain itself. PNE was not reported as significantly different from sham PNE groups regarding safety or adverse effects.
Rejas-Fernández et al. [56]	Pain intensity (NPRS): Ultrasound-guided percutaneous electrolysis (PE) added to manual therapy and exercise produced significantly greater pain reduction than manual therapy and exercise alone at all follow-ups: post-treatment (Δ −3.4, 95% CI −4.8 to −2.0), 3 months (Δ −3.4, 95% CI −5.0 to −1.8), and 6 months (Δ −2.6, 95% CI −4.2 to −1.0).	Disability (Foot and Ankle Ability Measure, FAAM): The PE group showed significantly greater improvement than the comparator group in both FAAM-Sports and FAAM-ADL at all assessed time points. For post-treatment, FAAM-Sports Δ 49.6 (95% CI 34.6 to 64.6) and FAAM-ADL Δ 26.9 (95% CI 14.6 to 39.2); for 3 months, FAAM-Sports Δ 31.6 (95% CI 14.8 to 48.4) and FAAM-ADL Δ 4.9 (95% CI 2.0 to 7.8); for 6 months, FAAM-Sports Δ 17.4 (95% CI 2.0 to 32.8) and FAAM-ADL Δ 2.4 (95% CI 1.0 to 3.8).

**Table 6:** Summary of the main findings for Percutaneous Needle Electrolysis (PNE) in other conditions.

Study	Main Findings from Primary Outcomes	Main Findings from Secondary Outcomes
Miguel-Valtierra et al. [40] Subacromial pain syndrome	Functional Improvement (DASH): The inclusion of Ultrasound (US)-guided PNE into manual therapy and exercise showed no significant differences for related disability (Disabilities of the Arm, Shoulder and Hand—DASH) at any follow-up (*p* = 0.051). PNE was not significantly better than manual therapy and exercise alone in overall shoulder-related disability (DASH). Functional Improvement (SPADI): US-guided PNE showed significantly greater changes in function (Shoulder Pain and Disability Index—SPADI) (*p* < 0.001) with large effect sizes compared to manual therapy and exercise alone at all follow-ups. PNE was significantly better than manual therapy and exercise alone in shoulder function (SPADI).	Pain Intensity (Shoulder Pain): US-guided PNE showed significantly greater changes in shoulder pain (*p* < 0.001) with large effect sizes compared to manual therapy and exercise alone at all follow-ups. PNE was significantly better than manual therapy and exercise alone in mean, worst, and least shoulder pain intensity. Pressure Pain Thresholds (PPTs): No statistically significant between-group differences in PPTs were found at any location. PNE was not significantly better than manual therapy and exercise alone in Pressure Pain Thresholds (PPTs). Patient-Reported Outcomes (GROC): Significantly more patients in the US-guided PNE group achieved a successful outcome based on Global Rating of Change (GROC) at 3 (*p* = 0.006) and 6 (*p* < 0.001) months. PNE was significantly better than manual therapy and exercise alone in the proportion of patients achieving a successful outcome (GROC). Safety/Adverse Effects: Muscle soreness (24%) was experienced in the PNE group, resolving spontaneously within 24–36 hours. Comparative differences in safety and adverse effects were not explicitly reported as significant.
Fernández-Rodríguez et al. [46] Chronic painful heel pain/plantar fasciopathy	Pain Intensity (NPRS): Ultrasound-guided PNE significantly better than placebo puncture in pain (Numerical Pain Rating Scale—NPRS) at 1, 12, and 24 weeks (*p* < 0.001). PNE was significantly better than placebo puncture in pain intensity (NPRS). Functional Improvement (FAAM ADL): Ultrasound-guided PNE significantly better than placebo puncture in function and disability (Foot and Ankle Ability Measure Activities of Daily Living Subscale—FAAM ADL) at 1, 12, and 24 weeks (*p* < 0.002). PNE was significantly better than placebo puncture in functional improvement (FAAM ADL).	Fascia Thickness: Ultrasound-guided PNE showed a significant decrease in plantar fascia thickness at 24 weeks compared to baseline, but the authors could not conclude true improvements. PNE was significantly better than placebo puncture in decreasing fascia thickness, though this effect was not definitively confirmed as a true biological change by the authors. Safety/Adverse Effects: No adverse events were reported in association with the trial intervention. PNE was not significantly different from placebo puncture regarding the occurrence of adverse events.
Rodríguez-Huguet et al. [47]	Pain Intensity (NPRS): PNE significantly better than Trigger Point Dry Needling (TDN) in pain intensity (Numerical Pain Rating Scale—NPRS) at post-treatment (*p* < 0.001), 1-month (*p* < 0.001), and 3-month (*p* < 0.001) follow-ups. PNE was significantly better than TDN in pain intensity (NPRS). Functional Improvement (ROM): PNE significantly better than TDN in elbow flexion Range of Motion (ROM) at post-treatment (*p* = 0.006), 1-month (*p* = 0.036), and 3-month (*p* = 0.003) follow-ups. PNE was not significantly better than TDN in elbow Extension, Supination, or Pronation ROM.	Quality of Life (SF-12): PNE was not significantly better than TDN in quality of life (Mental Component Score—MCS-SF12, Physical Component Score—PCS-SF12 of the SF-12 questionnaire). Safety/Adverse Effects: No significant comparative differences in safety or adverse effects were explicitly reported.
Moreno et al. [48] Adductor-related groin pain	Pain Intensity (VAS): Electrolysis Percutaneous Intratissue (EPI^®^), a form of PNE, in all intervention groups (Trigger Points—TP, Tendon—T, Trigger Points and Tendon—TTP) significantly reduced perceived pain (Visual Analogue Scale—VAS) compared to the Control Group (CT) at all measured time points (*p* < 0.001). The TTP group showed the best results. PNE (TTP group) was significantly better than PNE (T group) and PNE (TP group) in pain reduction. PNE (T group) and PNE (TP group) were not significantly different in pain reduction after two applications.	Safety/Adverse Effects: No specific adverse events or their statistical comparison between groups were reported. PNE (EPI^®^) was not reported as significantly different from control regarding safety or adverse effects.
Al-Boloushi et al. [43] Chronic painful heel pain/plantar heel pain	Pain Intensity (Foot Pain domain): Percutaneous Needle Electrolysis (PNE) and Dry Needling (DN) both significantly improved the Foot Pain domain of the Foot Health Status Questionnaire (FHSQ) at all time points (*p* < 0.001). PNE was not significantly better than DN in the Foot Pain domain. Pain Intensity (VAS): PNE and DN both significantly decreased Numerical Rating Scale Pain Visual Analogue Scale (VAS) scores at all time points (*p* < 0.001). PNE was not significantly better than DN in VAS average pain at 4 weeks. PNE was not significantly better than DN in VAS maximum pain at 4 weeks. (Note: DN showed benefit over PNE for VAS average (*p* = 0.009) and VAS maximum (*p* = 0.043) at 4 weeks). Functional Improvement (Foot Function domain): PNE and DN both significantly improved the Foot Function domain of FHSQ (*p* < 0.001). PNE was not significantly better than DN in the Foot Function domain. Functional Improvement (Footwear, GFH domains): PNE and DN both significantly improved Footwear and General Foot Health (GFH) domains of FHSQ (*p* = 0.031 and *p* < 0.001 respectively for DN; *p* < 0.001 for both for PNE). PNE was not significantly better than DN in the Footwear domain or the General Foot Health (GFH) domain.	Quality of Life (QoL): PNE and DN both improved Quality of Life (EuroQoL-5 dimensions—EQ-5D-5L) at 4 weeks. PNE showed improvements at 8 weeks and 52 weeks. PNE was significantly better than DN in Quality of Life (QoL) at 52 weeks (*p* < 0.05). Safety/Adverse Effects: Two small hematomas were reported in the PNE group and one in the DN group; no serious adverse events occurred. PNE was not significantly different from DN regarding the occurrence of reported minor adverse events.
León-Hernández et al. [38] Chronic non-specific neck pain	Pain Intensity (NPI): Percutaneous Electrical Nerve Stimulation (PENS), a form of IMES, combined with Dry Needling (DN + PENS) showed significant between-group differences in Neck Pain Intensity (NPI) compared to DN alone immediately after treatment (*p* < 0.05). IMES (PENS) was significantly better than DN alone in neck pain intensity (NPI) immediately after treatment. Functional Improvement (CROM): No statistically significant changes were observed in any cervical Range of Motion (CROM) movements for the time × group interaction (*p* > 0.05 in all), meaning no differences between groups were found. IMES (PENS) was not significantly better than DN alone in cervical Range of Motion (CROM). Functional Improvement (NDI): No significant between-group differences in Neck Disability Index (NDI) were found at 72 h post-treatment (*p* > 0.05). IMES (PENS) was not significantly better than DN alone in neck disability (NDI).	Pain Intensity (PNS): IMES (PENS) combined with DN showed significant between-group differences in post-needling soreness (PNS) compared to DN alone at all follow-up periods (*p* < 0.05). IMES (PENS) was significantly better than DN alone in post-needling soreness (PNS). Pressure Pain Threshold (PPT): IMES (PENS) combined with DN showed a higher improvement in Pressure Pain Threshold (PPT) compared to DN alone immediately post-DN (*p* < 0.05). IMES (PENS) was significantly better than DN alone in Pressure Pain Threshold (PPT) immediately post-DN. Safety/Adverse Effects: No adverse events were reported in the provided text. IMES (PENS) was not reported as significantly different from DN alone regarding safety or adverse effects.
Pérez-Palomares et al. [32] Nonspecific chronic low back pain	Pain Intensity (VAS): The improvement in perceived pain (Visual Analog Scale—VAS) was similar for both Percutaneous Electrical Nerve Stimulation (PENS) and Dry Needling (DN) groups (*p* = 0.94). PENS was not significantly better than DN in perceived pain (VAS). Functional Improvement (Oswestry Disability Index—other sections): No significant differences were found between PENS and DN therapies for other sections of the Oswestry Disability Index (personal care *p* = 0.94, walking *p* = 0.86, sitting *p* = 0.51, standing *p* = 0.26, social life *p* = 0.18). PENS was not significantly better than DN in other sections of the Oswestry Disability Index.	Quality of Life (Oswestry Disability Index—“lifting weight”): Improvement in “lifting weight” was significantly greater for the Dry Needling (DN) technique compared to Percutaneous Electrical Nerve Stimulation (PENS) (*p* = 0.03). PENS was not significantly better than DN in the “lifting weight” section of the Oswestry Disability Index. Sleep Quality (VAS): The improvement in sleep quality (Visual Analog Scale—VAS) was similar for both PENS and DN groups (*p* = 0.68). PENS was not significantly better than DN in sleep quality (VAS). Safety/Adverse Effects: Limited adverse effects were mentioned generally for dry needling as a useful tool, but no specific comparative adverse events were reported for PENS vs. DN in this study. PENS was not reported as significantly different from DN regarding safety or adverse effects.

**Table 7:** Summary of the main findings for intramuscular electrical stimulation IMES in myofascial pain/trigger point.

**Study**	**Main Findings from Primary Outcomes**	**Main Findings from Secondary Outcomes**
Byeon et al., 2003 [33]	Pain Intensity (VAS): Intramuscular Stimulation (IMS) showed significant reduction in Visual Analogue Scale (VAS) at 1 week compared to Dry Needling (DN) and Intramuscular Electrical Stimulation (IMES) (*p* < 0.05). IMS was significantly better than DN and IMES in VAS from 1 week after treatment. Functional Improvement (PROM): IMS showed significant increase in cervical Passive Range of Motion (PROM) from 3 days after treatment, while DN and IMES did not show significant increase. IMS was significantly better than DN and IMES in cervical Passive Range of Motion (PROM) from 3 days after treatment.	Pain Intensity (MPQ): All groups showed significant within-group reduction in McGill Pain Questionnaire (MPQ) scores from 2 weeks after treatment. IMS was not significantly different from DN or IMES in McGill Pain Questionnaire (MPQ) scores. Quality of Life (QoL): Not reported in the provided text. IMS was not reported as significantly different from DN or IMES in Quality of Life (QoL). Safety/Adverse Effects: Not explicitly reported in the provided text for this study. IMS was not reported as significantly different from DN or IMES regarding safety or adverse effects. Safety/Adverse Effects: Not explicitly reported in the provided text for this study. IMS was not reported as significantly different from DN or IMES regarding safety or adverse effects.
Sumen et al. [50]	Pain Intensity (VAS): Intramuscular Electrical Stimulation (IMS) and Low-Level Laser Therapy (LLLT) both showed significantly lower pain scores (Visual Analogue Scale—VAS) compared to the stretching exercise control group at one month after treatment (IMS: *p* = 0.001; LLLT: *p* = 0.016). IMS was not significantly different from LLLT in pain intensity (VAS) at any evaluated time point. Functional Improvement (NDI, ROM): IMS and LLLT both showed significant within-group improvements in Neck Disability Index (NDI) and cervical Range of Motion (ROM). IMS was not significantly different from the stretching exercise control group in NDI or ROM. LLLT was not significantly different from the stretching exercise control group in NDI or ROM. IMS was not significantly different from LLLT in NDI or ROM at any evaluated time point.	Pain Threshold (PT): IMS showed significantly higher pain threshold scores (PT) compared to the stretching exercise control group at one month after treatment (*p* = 0.017). LLLT did not show a significantly higher pain threshold compared to the stretching exercise control group. IMS was not significantly different from LLLT in pain threshold (PT) at any evaluated time point. Safety/Adverse Effects: Not explicitly reported in the provided text. IMS was not reported as significantly different from LLLT or the stretching exercise control group regarding safety or adverse effects.
Medeiros et al. [36]	Pain Intensity (VAS): Deep Intramuscular Stimulation Therapy (DIMST), a form of IMES, significantly reduced pain intensity (Visual Analogue Scale—VAS) compared to sham-DIMST both immediately before and after the intervention (*p* < 0.05). IMES (DIMST) was significantly better than sham-DIMST in pain intensity (VAS). Neuromuscular Performance (Synergistic Effect): There was no observed synergistic effect related to the association of rTMS and DIMST for pain reduction. IMES (DIMST) in combination with rTMS was not significantly better than individual components for synergistic effects.	Neurophysiological Parameters (MEP): No significant change in Motor Evoked Potentials (MEP) directly attributable to DIMST alone. There was a tendency for an increase in MEP with rTMS + DIMST (*p* = 0.08), but this was not statistically significant. IMES (DIMST) was not significantly better than sham-DIMST in neurophysiological parameters (MEP amplitude). Safety/Adverse Effects: No serious or moderate side effects were observed. IMES (DIMST) was not significantly different from sham-DIMST regarding safety or adverse effects. Biochemical Parameters: No significant changes were found in serum levels of BDNF, S100b, lactate dehydrogenase, inflammatory markers (TNF-α, IL-6, IL-10), or oxidative stress parameters. IMES (DIMST) was not significantly better than sham-DIMST in any peripheral biochemical parameters.
Hadizadeh et al. [51]	Pain Intensity (VAS): Intramuscular Electrical Stimulation (IMES) showed significant improvement in Visual Analogue Scale (VAS) pain scores one week after intervention compared to the placebo group (*p* = 0.048). IMES was not significantly different from placebo in VAS immediately after treatment. IMES was significantly better than placebo in pain intensity (VAS) one week after treatment. Functional Improvement (ROM): IMES showed significantly higher cervical Range of Motion (ROM) one week after treatment compared to the placebo group (*p* = 0.019). IMES was not significantly different from placebo in ROM immediately after treatment. IMES was significantly better than placebo in Range of Motion (ROM) one week after treatment.	Safety/Adverse Effects: Not explicitly reported in the provided text. IMES was not significantly different from placebo regarding the occurrence of adverse events.
Botelho et al. [19]	Pain Intensity (Daily Pain Scores): Intramuscular Electrical Stimulation (EIMS), a form of IMES, significantly decreased daily pain scores by −73.02% compared to the sham group at 3 months of follow-up (*p* < 0.001). IMES was significantly better than sham in daily pain scores. Pain Intensity (NPS during CPM-task): EIMS significantly reduced Numerical Pain Scale (NPS0-10) scores during the Conditioned Pain Modulation (CPM)-task compared to sham (mean difference −1.25, *p* = 0.01). IMES was significantly better than sham in NPS during the CPM-task. Functional Improvement (Disability due to pain—B-PCP:S): EIMS significantly improved disability due to pain (Brazilian Profile of Chronic Pain: Screen—B-PCP:S score) by −43.19% compared to the sham group at 3 months of follow-up (*p* < 0.0001). IMES was significantly better than sham in disability due to pain (B-PCP:S).	Safety/Adverse Effects: No severe or moderate side effects were observed. IMES was not significantly different from sham regarding the occurrence of severe or moderate side effects. Analgesic Use: The relative risk for using analgesics was 2.95 (95% CI, 1.36 to 6.30) in the sham group, indicating significantly lower analgesic use in the EIMS group (*p* < 0.01). IMES was significantly better than sham in reducing analgesic use. Neurophysiological Changes (MEP, SICI, ICF, CSP): EIMS significantly decreased Motor Evoked Potentials (MEP) by 28.79% (*p* = 0.02) and significantly increased Short Intracortical Inhibition (SICI) by 37.41% (*p* = 0.005) compared to sham. EIMS was not significantly different from sham in Intracortical Facilitation (ICF) or Current Silent Period (CSP). IMES was significantly better than sham in MEP and SICI. Sleep Quality (VAS-QS): EIMS improved previous night sleep quality (Visual Analogue Scale—Quality of Sleep—VAS-QS) by 12.75% compared to habitual sleep quality (*p* = 0.004). IMES was significantly better than sham in previous night sleep quality (VAS-QS). Neuroplasticity Markers (BDNF): A significant increase in serum Brain-Derived Neurotrophic Factor (BDNF) induced by EIMS predicted its long-term impact on chronic MPS symptoms. Higher baseline MEP amplitude and a greater increase in serum BDNF were predictors of EIMS effect on pain and disability. IMES was significantly better than sham in increasing serum BDNF.
Brennan et al. [35]	Pain Intensity (NPRS): Both Dry Needling (DN) and Dry Needling with Intramuscular Electrical Stimulation (DN/IMES) showed significant within-group improvement in pain (Numerical Pain Rating Scale—NPRS) between weeks 0–6 (DN: *p* = 0.017; DN/IMES: *p* = 0.018). DN/IMES was not significantly better than DN alone in pain intensity (NPRS) at week 6 or 12. Functional Improvement (NDI): Both DN and DN/IMES showed significant within-group improvement in disability (Neck Disability Index—NDI) between weeks 0–6 (DN: *p* = 0.008; DN/IMES: *p* < 0.001). DN/IMES was not significantly better than DN alone in disability (NDI) at week 6 or 12.	Safety/Adverse Effects: Three participants experienced a vasovagal response. IMES (combined with DN) was not significantly different from DN alone regarding the occurrence of vasovagal responses (adverse events).
Conti et al. [31]	Pain Intensity (PI): Contingent Electrical Stimulation (CES), a form of IMES, did not show significant differences in present pain intensity (PI) levels at any phase. IMES (CES) was not significantly better than the inactive device (control group) in present pain intensity (PI). Neuromuscular Performance (EMG/h): CES significantly reduced electromyographic events per hour of sleep (EMG/h) by 35% in Phase 2 (*p* = 0.004) and maintained a 38.4% reduction in Phase 3 compared to baseline. The mean EMG activity was lower in the active group than the control group in Phase 2. IMES (CES) was significantly better than the inactive device (control group) in electromyographic events per hour of sleep (EMG/h) in Phase 2, but not significantly better in Phase 3.	Pressure Pain Threshold (PPT): CES did not show significant differences in pressure pain threshold (PPT) levels at any phase. IMES (CES) was not significantly better than the inactive device (control group) in pressure pain threshold (PPT).
Shanmugam et al. [52]	Pain Intensity (VAS): Intramuscular Electrical Stimulation (IMES) using both Inverse Electrode Placement (IEP) and Conventional Electrode Placement (CEP) significantly reduced upper trapezius (UT) pain severity (Visual Analogue Scale—VAS) compared to sham-IMES at day three follow-up (*p* = 0.001 for IEP, *p* = 0.007 for CEP). IMES (IEP) and IMES (CEP) were significantly better than sham-IMES in reducing pain severity (VAS). IMES (IEP) was not significantly better than IMES (CEP) in reducing pain severity (VAS). Functional Improvement (ROM): IMES (both IEP and CEP) significantly improved upper trapezius (UT) muscle length (Range of Motion—ROM) compared to sham-IMES at day three follow-up (*p* < 0.001 for IEP, *p* < 0.001 for CEP). IMES (IEP) and IMES (CEP) were significantly better than sham-IMES in improving UT muscle length (ROM). IMES (IEP) was not significantly better than IMES (CEP) in improving UT muscle length (ROM).	Pressure Pain Threshold (PPT): IMES (both IEP and CEP) significantly increased upper trapezius (UT) Pressure Pain Threshold (PPT) compared to sham-IMES at day three follow-up (*p* < 0.001 for IEP, *p* < 0.001 for CEP). IMES (IEP) and IMES (CEP) were significantly better than sham-IMES in increasing UT Pressure Pain Threshold (PPT). IMES (IEP) was not significantly better than IMES (CEP) in increasing UT Pressure Pain Threshold (PPT). Neuromuscular Performance (EMG activity—RMS): IMES (both IEP and CEP) significantly reduced Root Mean Square (RMS) activity compared to sham-IMES at day three follow-up (*p* = 0.002 for IEP, *p* = 0.004 for CEP). IMES (IEP) and IMES (CEP) were significantly better than sham-IMES in reducing EMG (RMS) activity. IMES (IEP) was not significantly better than IMES (CEP) in reducing EMG (RMS) activity. Safety/Adverse Effects: No specific adverse events were reported for either group. IMES (IEP) and IMES (CEP) were not reported as significantly different from sham-IMES regarding safety or adverse effects.
Hadizadeh et al. [37]	Functional Improvement (ROM): Intramuscular Electrical Stimulation (IMES) showed significantly greater neck Range of Motion (ROM) increment compared to Dry Needling (DN) post-intervention and one month after intervention (*p* = 0.000). IMES was significantly better than DN in neck Range of Motion (ROM). Neuromuscular Performance (TrP Circumference): IMES showed significantly greater TrP circumference decrement compared to DN post-intervention and one month after intervention (*p* = 0.000). IMES was significantly better than DN in reducing TrP circumference. Neuromuscular Performance (TrP Longitudinal Diameter): IMES showed significantly greater TrP longitudinal diameter changes compared to DN post-intervention and one month after intervention (*p* = 0.000). IMES was significantly better than DN in TrP longitudinal diameter.	Pain Intensity (VAS): IMES did not show a significant difference in pain reduction (Visual Analogue Scale—VAS) between groups post-intervention or one month after intervention. IMES was not significantly better than DN in pain intensity (VAS). Pressure Pain Threshold (PPT): IMES showed significantly greater Pressure Pain Threshold (PPT) improvement compared to DN post-intervention and one month after intervention. IMES was significantly better than DN in Pressure Pain Threshold (PPT). Disability (NDI): IMES did not show a significant difference in Neck Disability Index (NDI) changes compared to DN. IMES was not significantly different from DN in neck disability (NDI). Safety/Adverse Effects: Safety and adverse effects were not explicitly reported as significantly different between groups.
Niddam et al. [53]	Pain Intensity (PT to IMES stimuli): Low-intensity intramuscular electrostimulation (IMES) led to a significant increase in pain tolerance (PT) after intervention for responders (10 of 21 patients, *p* = 0.041). IMES was significantly better than control (implied by responder analysis) in increasing pain tolerance (PT) for a subgroup of patients. Neuromuscular Performance (PAG activity): IMES intervention significantly modulated Periaqueductal Gray (PAG) activity to painful stimuli more in responders than in nonresponders (*p* = 0.009). Change in PAG activity correlated with change in pressure pain threshold (r = 0.455, *p* = 0.0382). IMES was significantly better than control (implied by responder analysis) in modulating PAG activity.	Pressure Pain Threshold (PPT): 12 of 21 patients showed a significant increase in post-intervention Pressure Pain Threshold (PPT) (*p* < 0.0001 for PPT-change between responders and nonresponders). IMES was significantly better than control (implied by responder analysis) in increasing Pressure Pain Threshold (PPT). Safety/Adverse Effects: Not explicitly reported in the provided text. IMES was not reported as significantly different from control regarding safety or adverse effects.
Ga et al. [55]	Pain Intensity (Wong-Baker FACES, VAS): Intramuscular Stimulation (IMS) resulted in a significant reduction in Wong-Baker FACES pain scale scores at all visits and was more effective than 0.5% lidocaine injection to trigger points (TPI). However, there was no significant between-group difference in Visual Analogue Scale (VAS) pain. IMS was significantly better than TPI in Wong-Baker FACES pain scale scores, but not significantly better in VAS pain. Functional Improvement (Passive Cervical ROM): All passive cervical Ranges of Motion (ROMs) significantly increased in both groups. IMS was significantly better than TPI only in extension of the ROM.	Patient-Reported Outcomes (GDS-SF): IMS resulted in significant improvement on the Geriatric Depression Scale—Short Form (GDS-SF) at the end of the first month after treatment (*p* = 0.024). IMS was significantly better than TPI in the Geriatric Depression Scale—Short Form. Safety/Adverse Effects: Post-treatment soreness was noted in 54.6% of the IMS group and 38.1% of the TPI group, with no significant differences in number or duration. Gross subcutaneous hemorrhage (>4 cm^2^) was seen in only one TPI patient. IMS was not significantly different from TPI regarding post-treatment soreness or hemorrhage. Pressure Pain Threshold (PTS): No significant pre- and post-treatment difference in trigger point pain pressure threshold scores (PTS) was found between both groups at all visits (*p* > 0.05). IMS was not significantly better than TPI in trigger point pain pressure threshold scores (PTS).
Hadizadeh et al. [45]	Pain Intensity (VAS): Intramuscular Electrical Stimulation (IMES) showed significantly lower pain (Visual Analogue Scale—VAS) one week after treatment compared to the placebo group (*p* < 0.05). IMES was significantly better than placebo in pain intensity (VAS) one week after treatment. Functional Improvement (ROM): IMES showed significantly greater cervical Range of Motion (ROM) immediately after and one week after the intervention compared to the placebo group (*p* < 0.05). IMES was significantly better than placebo in cervical Range of Motion (ROM) immediately after and one week after treatment.	Pressure Pain Threshold (PPT): IMES showed significantly higher Pressure Pain Threshold (PPT) one week after the intervention compared to the placebo group (*p* < 0.05). IMES was significantly better than placebo in Pressure Pain Threshold (PPT) one week after treatment. Disability (NDI): Disability in both groups decreased, but there was no significant difference between the IMES and placebo groups. IMES was not significantly different from placebo in neck disability (NDI). Safety/Adverse Effects: No adverse events were reported other than needle-related pain and post-puncture pain, which were outcome measures. IMES was not reported as significantly different from placebo regarding safety or adverse effects.
Zuccolotto Moro et al. [44]	Pain Intensity (VAS): Electroacupuncture of motor points and/or the spinal accessory nerve (Motor Point EA/SAN) showed significantly lower average pain levels (Visual Analogue Scale—VAS) compared to dry needling of trigger points (DN) across repeated assessments (mean difference = 0.98; *p* = 0.012). No significant differences in pain scores were found between DN and intramuscular electrical stimulation of trigger points (IMES of TrPs). Motor Point EA/SAN was significantly better than DN in pain intensity (VAS). IMES of TrPs was not significantly better than DN in pain intensity (VAS). Functional Improvement: Not explicitly reported as a primary outcome with comparative group differences beyond pain relief and quality of life.	Quality of Life (SF-12): No significant differences were found in quality of life (12-item Short Form health questionnaire—SF-12) across the three groups at the end of the treatment period. Motor Point EA/SAN was not significantly different from DN or IMES of TrPs in quality of life (SF-12). IMES of TrPs was not significantly different from DN in quality of life (SF-12). Safety/Adverse Effects: Not explicitly reported in the provided text.
Sharma et al. [34]	Pain Intensity (NPRS): Both dry needling with Intramuscular Electrical Stimulation (DN/IMES) at 2 Hz and 100 Hz frequencies showed statistically significant improvement in Numeric Pain Rating Scale (NPRS) (*p* = 0.001 and *p* = 0.00005, respectively), while the DN group showed no significant improvement (*p* = 0.726). DN/IMES [f-100 Hz] showed more significant improvement than DN/IMES [f-2 Hz] (*p* = 0.006). Both DN/IMES groups were significantly better than DN alone (*p* = 0.013 for DN/IMES [f-2 Hz] vs. DN; *p* = 0.000002 for DN/IMES [f-100 Hz] vs. DN). Functional Improvement: Not explicitly reported as a primary outcome with comparative group differences beyond pain/soreness related to trigger points.	Pressure Pain Threshold (PPT): Both DN/IMES at 2 Hz and 100 Hz frequencies showed statistically significant improvement in Pain Pressure Threshold (PPT) (*p* = 0.004 and *p* = 0.0002, respectively), while the DN group showed no significant improvement (*p* = 0.238). DN/IMES [f-100 Hz] was significantly better than DN/IMES [f-2 Hz] (*p* = 0.012) and DN alone (*p* = 0.000117). DN/IMES [f-2 Hz] was not significantly better than DN alone (*p* = 0.291). Safety/Adverse Effects: Not explicitly reported in the provided text.

## Data Availability

No new data were created or analyzed in this study.

## Supplementary Materials

The following supporting information can be downloaded at: https://www.mdpi.com/article/10.3390/jcm15072572/s1, PRISMA 2020 Checklist.

## Abbreviations

The following abbreviations are used in this manuscript:

ALErGP	Adductor-related groin pain
ADL	Activities of daily living
APT	Active physical therapy
AT	Achilles tendinopathy
BDNF	Brain-derived neurotrophic factor
CCT	Controlled clinical trial
CEP	Conventional electrode polarity
CES	Contingent electrical stimulation
CLBP	Chronic low back pain
CPHP	Chronic painful heel pain
CROM	Cervical range of motion
CSP	Cortical silent period
DASH	Disabilities of the Arm, Shoulder and Hand
DDN	Deep dry needling
DIMST	Deep intramuscular stimulation therapy
DN	Dry needling
EE	Eccentric exercise
EG	Experimental group
EIMS	Electrical intramuscular stimulation
EMG	Electromyography
EMG/h	Electromyographic events per hour
EPI	Electrolysis Percutaneous Intratissue
EQ-5D-5L	EuroQol 5-Dimension 5-Level
FAAM	Foot and Ankle Ability Measure
FHSQ	Foot Health Status Questionnaire
fMRI	Functional magnetic resonance imaging
GDS-SF	Geriatric Depression Scale-Short Form
GFH	General foot health
GROC	Global Rating of Change
HI-USGET	High-intensity ultrasound-guided galvanic electrolysis technique
HPT	Heat pain threshold
ICF	Intracortical facilitation
IEP	Inverse electrode polarity
IMES	Intramuscular electrical stimulation
IMS	Intramuscular stimulation
ICTRP	International Clinical Trials Registry Platform
LE	Lateral epicondylitis
LDH	Lactate dehydrogenase
LLLT	Low-level laser therapy
LPM	Lateral pterygoid muscle
MD	Mean difference
MEP	Motor evoked potentials
MEP (microelectrolysis)	Percutaneous microelectrolysis
MeSH	Medical Subject Headings
MIO	Maximum interincisal opening
MPS	Myofascial pain syndrome
MTrP/MTrPs	Myofascial trigger point(s)
NDI	Neck Disability Index
NPI	Neck pain intensity
NPRS	Numerical Pain Rating Scale
NRS	Numeric rating scale
OR	Odds ratio
PE	Percutaneous electrolysis
PENS	Percutaneous electrical nerve stimulation
PICOS	Population, Intervention, Comparator, Outcomes, Study Design
PI	Pain intensity
PNE	Percutaneous needle electrolysis
PNM	Percutaneous neuromodulation
PNS	Post-needling soreness
PPT	Pressure pain threshold
PRISMA	Preferred Reporting Items for Systematic Reviews and Meta-Analyses
PROs	Patient-reported outcomes
PSFS	Patient-Specific Functional Scale
PT	Patellar tendinopathy
PTS	Pain pressure threshold scores
QoL	Quality of life
rTMS	Repetitive transcranial magnetic stimulation
RCT/RCTs	Randomized controlled trial(s)
RoB 2	Risk of Bias tool for randomized trials
ROBINS-I	Risk Of Bias In Non-randomized Studies of Interventions
ROM	Range of motion
RR	Risk ratio
RMS	Root mean square
ROS	Reactive oxygen species
SF-12	Short Form-12 (health survey)
SF-36	Short Form-36 (health survey)
SICI	Short intracortical inhibition
SMD	Standardized mean difference
SNP	Sham needle puncture
SOD	Superoxide dismutase
SPADI	Shoulder Pain and Disability Index
TDN	Trigger point dry needling
TENS	Transcutaneous electrical nerve stimulation
TMJ	Temporomandibular joint
TPI	Trigger point injection
TrP/TrPs	Myofascial trigger point(s)
TNF-alpha	Tumor necrosis factor alpha
US	Ultrasound
USGET	Ultrasound-guided galvanic electrolysis technique
UT	Upper trapezius
VAS	Visual Analogue Scale
VAS-QS/VASQS	Visual Analogue Scale-Quality of Sleep
VISA-A	Victorian Institute of Sports Assessment-Achilles
VISA-P	Victorian Institute of Sports Assessment-Patellar
WHO	World Health Organization
